# Peruvian License Plate Recognition at the Edge: Compact LPRNet and CRNN Recognizers Distilled from a Rule-Corrected Generic OCR

**DOI:** 10.3390/s26217076

**Published:** 2026-11-09

**Authors:** Angel Rosendo Condori-Coaquira, Esteban Tocto-Cano, Milton Edward Humpiri-Flores, David Mamani-Pari, Godofredo Quea-Lopez

**Affiliations:** Escuela Profesional de Ingeniería de Sistemas, Facultad de Ingeniería y Arquitectura, Universidad Peruana Unión, Juliaca 21101, Perumilton.humpiri@upeu.edu.pe (M.E.H.-F.); davidmp@upeu.edu.pe (D.M.-P.);

**Keywords:** automatic license plate recognition, optical character recognition, model distillation, weakly supervised learning, CTC, LPRNet, CRNN, edge computing, Peru

## Abstract

High recognition rates reported for automatic license plate recognition (ALPR) rarely survive deployment. We analyze a fixed-camera edge ALPR system that ran without interruption for 465 days at the vehicle entrance of a university on the Peruvian altiplano and logged 579,292 crops from 29,928 unique plates over 294,900 vehicle passes. Because the camera is mounted away from the lane, the median plate measures 101 px wide by 51 px high, a resolution at which character segmentation is no longer reliable and the whole string must be read at once. The pipeline is a two-stage one. A YOLOv8n detector locates the plate on the full frame at 1.1 ms per image; the native crop is then read by PaddleOCR PP-OCRv4 reinforced with a deterministic corrector that encodes the Peruvian LNN-DDD pattern, and that pairing acts as a weak teacher producing silver labels at corpus scale. From those labels, we distill two compact recognizers on a unified 32 × 100 px input, LPRNet (0.97 M parameters, purely convolutional) and CRNN (8.73 M, two additional bidirectional recurrent layers), both trained with connectionist temporal classification (CTC) loss over 37 classes and decoding in parallel without character segmentation, and both fine-tuned on 1850 crops verified against the official vehicle registry. How the evaluation set is composed decides the conclusion. On 782 crops sampled by logged access, the teacher reached 95.4% and the student 97.4%, whereas on 1130 crops of arbitrary plates, the teacher collapsed to 54.7% while the student held 91.9% (+37.2 points; *p* = 7.4 × 10^−93^). The measured cause is that automatic label reliability grows with the number of times a vehicle passes the gate, rising from 40.4% on plates seen once or twice to 87.6% on those seen fifty times or more, a dependency the distilled model does not inherit. A rule-corrected optical character recognition (OCR) engine is, therefore, sufficient as a teacher for distilling edge-ready recognizers, and stating the sampling unit of the evaluation set is a precondition for interpreting any ALPR metric.

## 1. Introduction

Automatic license plate recognition (ALPR) has become a routine component of intelligent transportation systems. It controls access, collects tolls, manages parking, supports traffic enforcement and feeds public-safety workflows [1,2]. The task splits into two stages, locating the plate and reading its text, and deep learning has displaced hand-crafted pipelines in both [3,4].

Fixed-camera access control makes demands that differ from tolling or from capture aboard a moving vehicle. The sensor does not move; it runs for years without interruption, and it has to resolve the reading without stopping the incoming flow. Several recent studies describe installations of this kind on university and residential premises [5,6,7,8], some of them running recognition at the edge so that video never leaves the perimeter and latency stays bounded [9,10,11]. The siting involves a design tension: moving the camera away from the lane avoids intruding on traffic, but it also reduces the plate to a few tens of pixels.

Published accuracy does not survive deployment, and it fails in two ways that are seldom told apart. Models degrade when inference happens outside the environment they were trained in [12], so the metrics of one installation do not anticipate those of another. Published figures are also hard to compare with one another; a recent survey traces that opacity to the fact that every study partitions its data, evaluates, preprocesses, defines the task and configures its hardware in its own way [3].

Four causes converge. Size is the most obvious: as the camera moves back, the plate occupies fewer pixels and generic recognizers degrade, which has motivated the recourse to super-resolution as a palliative [13,14]. Next comes the character ambiguity carried by each national format. Pairs such as O/0 and I/1 are visually identical and are resolved only by attending to position within the pattern or, failing that, by consulting the official registry; the resulting error is systematic rather than random. The third cause is economic. Manual annotation costs money, hence the interest in knowledge distillation and weak supervision, which allow compact models to be trained without labeling at scale [15,16]. The fourth is that almost every proposal is tailored to one configuration or one plate region, which narrows its applicability [17].

The consequences compound. A test set built with the same optics and the same site as the training set yields optimistic estimates that do not hold once the installation changes. Protocol differences make fair architectural comparison across publications impossible and lead authors to credit the architecture for what actually comes from the dataset or the split [3,18]. Operationally, the cost is higher still: the true accuracy of a system is discovered once it is already in production.

A resource gap remains as well. No public ALPR dataset of Peruvian plates exists, nor of the Andean region, whose high altitude, intense radiation and harsh lighting contrast differ from the conditions represented by the available resources. Nor is there, as far as our review reaches, a South American dataset captured in prolonged continuous operation rather than in short campaigns.

This work sets out to design, deploy and evaluate a complete edge ALPR system for fixed-camera access control, and to establish under which evaluation conditions the claim holds that a compact distilled recognizer outperforms the generic optical character recognition (OCR) engine that produced its labels. The contributions are listed below in decreasing order of importance. The first is the core result of this study; the second and third follow from it; the remaining three are the resources and measurements that make the first three verifiable.

- Core contribution. The composition of the evaluation set determines the conclusion, and the mechanism behind it is measured rather than conjectured. Sampling by logged access or by distinct plate shifts the student’s margin over the teacher from 2.0 to 37.2 points on the same system and the same images. The cause is that the reliability of the automatic label grows with the number of vehicle passes, because labels consolidate by consensus across visits; the distilled model does not inherit that dependency. Both ground truths are released so that third parties can reproduce the comparison under either view.
- Evidence that a distilled and fine-tuned recognizer outperforms its own teacher on arbitrary plates, which establishes that a rule-corrected generic OCR engine is a sufficient teacher for weak supervision in this setting.
- Evidence that comparing architectures without equalizing the input resolution can reverse the conclusion. With the input unified and training repeated across three seeds, the purely convolutional network leads the convolutional recurrent one under both supervision regimes, with nine-times fewer parameters. This qualifies architectural comparisons that leave preprocessing uncontrolled [18].
- A sensing and edge processing system described in enough detail to be replicated, operated continuously for 465 days, from which 579,292 plate crops corresponding to 29,928 Peruvian plates are released under a use agreement with their own DOI. No public ALPR dataset of Peruvian or Andean plates existed previously.
- A deterministic position-aware corrector that encodes the national convention and acts as a cheap teacher for weak supervision, whose actual reach is quantified against two verified ground truths rather than assumed.
- A measurement of the cross-optics gap of the detection subsystem and its mitigation through a merged dataset, with the corresponding statistical protocol.

The remainder of this paper is organized as follows. Section 2 positions the work against the literature. Section 3 lays out the proposed methodology: the sensor, the acquisition chain, the datasets, the models compared and the statistical analysis. Section 4 presents the experiments, and Section 5 discusses them. Section 6 gathers the limitations of this study, and Section 7 concludes.

## 2. Related Work

### 2.1. Datasets and Geographical Bias

Progress in the field has been driven by public datasets [19], yet those datasets concentrate in a few regions. Their typefaces, formats and ambient conditions do not represent the rest. Efforts to widen coverage have looked to Europe [19] and to specialized domains such as vessels [20]; Latin America barely appears, and what little exists concentrates in Brazil [9,17]. A second shortcoming compounds the first, flagged in a recent survey: the limitations of the benchmarks themselves and the disparity of protocols make it difficult to establish how much real progress has been made [3]. We contribute a dataset from an unrepresented region, and we state the sampling protocol of the evaluation, whose omission is part of that second problem.

### 2.2. License Plate Detection

The YOLO family dominates localization thanks to its accuracy–latency balance [21,22,23], and it offers lightweight variants aimed at mobile and embedded devices [24,25]. Other proposals depart from the classical supervised scheme: end-to-end contrastive detectors [26] or knowledge distillation to cut computation without losing accuracy [15,16]. For oblique views, detecting the four corners allows the plate to be rectified to a fronto-parallel view before reading [17]. These components are adopted as established practice rather than reformulated. What the literature does lack is a measure of how much of that performance survives a change of optics, something that is acknowledged [12] but rarely quantified.

### 2.3. Text Recognition

Two families dominate. Sequence networks with connectionist temporal classification (CTC) loss, CRNN and its variants, avoid character segmentation and remain the reference [27,28,29], with compact versions designed for the edge [30,31]. Attention decoders and transformer-based models have gained ground against them [32,33,34]. A recent comparative study, carried out under identical detection conditions, asks whether the observed differences stem from sequence modeling or from the backbone representation [18]. Our results add a variable to that question: the resolution at which the crop is normalized is by itself enough to reverse the ordering between two architectures, so a comparison that does not equalize it measures preprocessing as much as it measures the model.

### 2.4. Adverse Conditions and Small Plates

Low resolution is the standard regime for cameras placed at a distance, and it has driven the systematic recourse to super-resolution as a preprocessing step [13,14,35], alongside the restoration of motion-degraded images [36]. Other lines attack poor illumination and occlusion [1,37]. The approach taken here differs: rather than restoring the image, we transfer to the compact recognizer the robustness that the corrected teacher already exhibits in that difficult regime. Dependence on plate size vanishes, and it does so without post-processing.

### 2.5. Edge Deployment and Access Control

Execution on resource-constrained devices has drawn growing attention: quantization and dedicated accelerators [9,10], efficient memory reuse [38], lightweight end-to-end architectures [11,39]. For access control, systems have been described on university and residential premises [5,6,7,8]. Nearly all of them evaluate the installation over short campaigns. Here, we document 465 consecutive days of operation, and we examine what happens to the metrics once the system faces the real population of plates instead of a convenience sample.

## 3. Proposed Methodology

### 3.1. Overview

**Figure 1:**
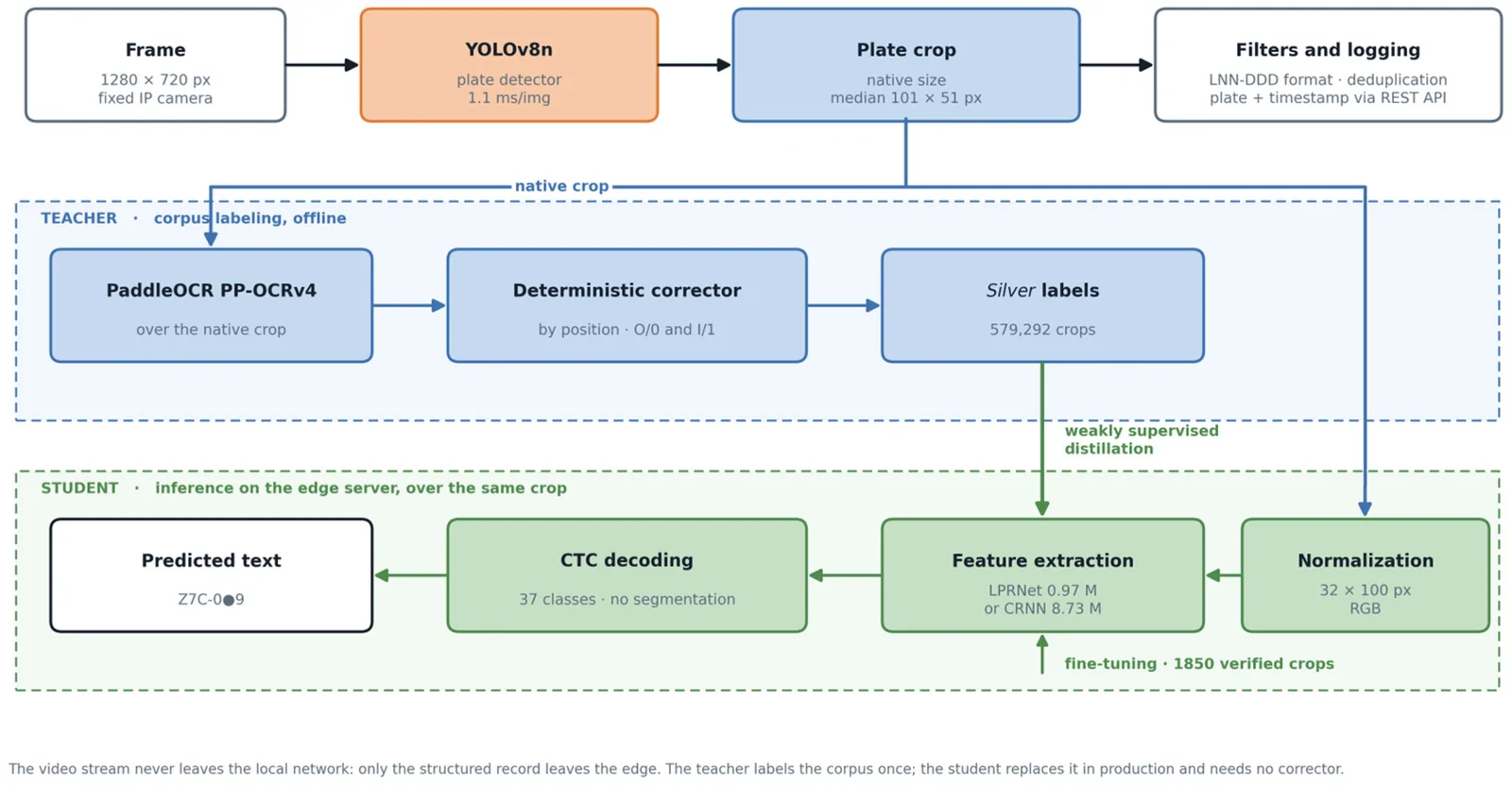
System architecture. Top, the capture chain: the frame passes through the detector and the plate is cropped at its native size. That crop feeds the two branches. The teacher (blue) reads it with a general-purpose optical character recognition (OCR) engine corrected by position and labels the corpus once, offline. The student (green) is distilled from those silver labels, fine-tuned on a verified set, and is the component deployed on the edge server without needing the corrector at inference time. Normalization to 32 × 100 px belongs to this second branch alone, since the compact recognizers require a fixed-size input. Colour encodes the role of each stage: orange for detection, blue for the teacher path, green for the student path, and white for the stages that carry no trainable parameters, namely the input frame, the filtering and logging that closes the chain, and the predicted string. The video stream never leaves the local network: only the structured record leaves the edge. One character is masked in the reading example, as elsewhere in this paper.

The methodology is organized into two chained phases. The first locates the plate within the frame; the second reads its characters. Figure 1 traces the complete path, from the video stream to the structured record.

That distinction is substantive rather than organizational, because the two phases play different roles here. Detection is the tool with which the dataset was built: it performs with a wide margin, poses no open problems, and we propose no new architecture for it. The residual error of the system lies in character recognition, where the contribution of this work is concentrated. Section 3.3, therefore, presents detection as a capture and labeling procedure, while Section 3.4 develops recognition as the object of study.

### 3.2. Study Design

This is an applied, explanatory study. It does more than describe how a system performs: it manipulates factors and tests their effect on a measured response. The design is factorial with repeated measures, so every experimental unit receives all the treatments compared.

- Experimental unit: the license plate, represented by one crop. Not the bare frame, since a single vehicle appears in dozens of near-identical captures, and not the physical vehicle, whose identity cannot be observed reliably. Under the by-plate view, the correspondence is nearly one to one (Section 3.3.6); under the by-event view, it deliberately is not.
- Observation unit: the (plate, system) pair.
- Response variable: exact match between the reading and the official registry, binary in nature. Character error rate (CER) serves as a secondary response.
- Factors: recognition system (6 levels, within-subject), lighting class (3 levels, between-subjects) and plate pass frequency (4 levels, between-subjects).

What is longitudinal here is the capture, 465 consecutive days that contribute seasonal and photometric variation. The comparison between systems, by contrast, is cross-sectional: it runs on fixed evaluation sets.

### 3.3. Phase 1: Dataset Construction

#### 3.3.1. Site and Sensing System

The system operates at the vehicle entrance of a university on the Peruvian altiplano, in Juliaca, Puno, above 3800 m a.s.l. The site is one of intense solar radiation, harsh lighting contrast and pronounced seasonality. The entrance carries continuous flow and does not allow vehicles to be stopped for the plate to be read.

The sensor is a Hikvision DS-2CD2683G2-LIZS2U 8 MP IP camera with motorized varifocal optics. It is mounted 2.50 m above the lane, aimed at the lower front area of the vehicle at an angle below 45° to the horizontal. The field of view centers on the plate: windshield and cabin fall outside the frame by design. Table 1 collects the specifications of the sensing subsystem and of the capture.

The sensor supports 8 MP, but the system works at 1280 × 720 px. This is not an instrument limitation but a decision: at native resolution, real-time processing introduced a delay incompatible with the access flow. The consequence conditions everything else and is worth stating outright. The median plate area is 5136 px^2^ and its median dimensions are 101 px wide by 51 px high, squarely within the small-object regime typical of cameras placed at a distance [13].

The site conditions deserve a fuller account, because they are what the recognizer actually faces. Juliaca lies at 3838 m above sea level and has a subtropical highland climate (Koppen Cwb) bordering on alpine tundra, with roughly 610 mm of annual precipitation concentrated in a wet season that runs from December to March. The dry season from June to August frequently records no precipitation at all. Two consequences matter for an outdoor vision system. The diurnal temperature range is large, with freezing nights and mild afternoons, so the enclosure and the optics work across a wide thermal span within a single day. And the combination of thin atmosphere and high solar elevation produces intense direct radiation, which at midday casts hard shadows across the plate and, on the specular surface of a retroreflective plate, produces saturation that no exposure setting removes entirely.

The camera operates continuously under all of these conditions; it is neither sheltered nor switched off during rain or hail. We did not instrument the site with a weather station, so the corpus carries no per-frame meteorological annotation, and we, therefore, make no claim about accuracy under rain as opposed to clear sky. What the corpus does carry, exactly, is the timestamp of every capture, which allows the seasonal and diurnal coverage to be stated without ambiguity (Section 3.3.4). Lighting is derived from true solar elevation at the site coordinates rather than from a clock cutoff, which matters at this latitude because civil twilight is short and a fixed hourly rule would misclassify a substantial share of captures.

#### 3.3.2. Plate Detection and the Edge Processing Chain

Localization uses YOLOv8n, for two reasons. It is the most widely adopted variant in the ALPR literature [21,22,23], and it is the fastest of those evaluated at 1.1 ms per image, which matters on an edge server handling continuous flow. To verify that the choice cost no accuracy, we trained ten architectures under identical conditions: YOLOv8, YOLOv11 and YOLOv12 in their nano, small and medium variants, plus RT-DETR. All of them reach a mean average precision at the 0.50 overlap threshold (mAP@50) between 0.994 and 0.995 and recall = 1.00 on the manually annotated test set, so latency becomes the deciding criterion. Section 4.6 reports the comparison in full.

The detector was trained on 2600 images with hand-annotated boxes, all from the same installation. The original partition of that set was made by file and at random, which turned out to leak the following: 54.0% of the images shared a plate string with another partition and 50.0% shared a temporal burst, taking a burst to be captures separated by five seconds or less. We, therefore, re-partitioned the set by entity, applying the same principle used for recognition in Section 3.3.5. Images are grouped by union of two criteria, identical plate string and membership in the same temporal burst, and a group is assigned whole to one partition. The result is 1820/390/390 images with no plate and no burst crossing a partition boundary. All detection figures reported in Section 4.6 come from this grouped split; the script that produces it and audits both partitions is released with the code.

The video stream is processed on an edge server located on the same local network, which keeps images inside the institutional perimeter. The chain has five stages:

1. Plate detection over the full frame.
2. Cropping tight to the detected box.
3. Text recognition through a general-purpose OCR engine.
4. Cascaded filters: deduplication by plate, national-format validation and a minimum crop area.
5. Logging, by sending the structured result over a REST API to the management application.

Only the structured record leaves the edge: plate string, timestamp and detection metadata. Privacy is thereby resolved at the source, and the latency constraints are met along the way, in line with embedded-execution proposals for ALPR [9,10].

#### 3.3.3. Automatic Labeling: Generic OCR and Deterministic Corrector

Every crop is read with PaddleOCR PP-OCRv4 [40], a general-purpose OCR engine with no domain adaptation. Its output then passes through a deterministic position-aware corrector that encodes the national convention. The Peruvian LNN-DDD pattern fixes which positions admit a letter and which a digit, and that alone resolves the O/0 and I/1 ambiguities that the image by itself cannot distinguish.

We call the concatenation of the two the teacher. It should be emphasized that this is not a trained model but a procedure: a generic OCR engine combined with a small set of rules, without a single parameter fitted to the data. It serves two functions. In operation, it produces the reading that is logged; at corpus scale, it produces the silver labels from which the compact recognizers are later distilled (Section 3.4.3). In the sense of weak supervision [15,16], it is an inexpensive teacher, since no manual annotation is required to obtain hundreds of thousands of image–text pairs.

Two limits of this device should be stated before the results rather than after them. A positional rule repairs a character whose identity is ambiguous but whose position is known; it cannot repair a character that was simply misread, so a PaddleOCR output of ‘B’ where the plate carries ‘8’, or ‘E’ where it carries ‘F’, passes through the corrector untouched and enters the silver labels as noise. And the rule set presumes the LNN-DDD pattern, so plates that depart from it, including motorcycle, diplomatic and special-series plates, are not corrected but discarded by the format filter. Neither limit is left as an assumption: Section 4.3.1 benchmarks the teacher’s pseudo-labels against both human-verified ground truths, and the resulting error rate, 4.6% by logged access and 45.3% by plate, provides the basis for the remainder of the analysis.

A consensus mechanism operates as well, and it is the source of the central result of this study and is, therefore, stated precisely here. The 579,292 retained crops correspond to 294,900 distinct vehicle passes: deduplication keeps a median of one crop per pass, with a mean of 2.0. Because the same vehicle crosses the entrance repeatedly over the period, the label of a plate consolidates across those passes, which improves the label of habitual vehicles and leaves untouched the label of anyone who passes once. The population is skewed towards the latter: 63.2% of plates were seen in two passes or fewer. Whatever the consensus contributes is, therefore, unavailable for most of the population, and Section 4.3.4 quantifies exactly how much accuracy that costs.

#### 3.3.4. Population, Filters and Resulting Dataset

The target population comprises the plates of four-wheel vehicles entering the campus, and the accessible population is the set the system logged during the operating period: 579,292 frames from 29,928 unique plates. Detector confidence on those crops starts at 0.60, the acceptance threshold, with a median of 0.92. As far as our review reaches, no public dataset of Peruvian or Andean plates exists at a comparable scale, nor one captured in prolonged continuous operation rather than in short campaigns.

The camera framing excludes vehicle occupants by design, but we nonetheless ran an automatic face sweep over the entire corpus. It detected identifiable pedestrians in 6357 frames, 1.10% of the total, and all of them were blurred before any further processing.

**Figure 2:**
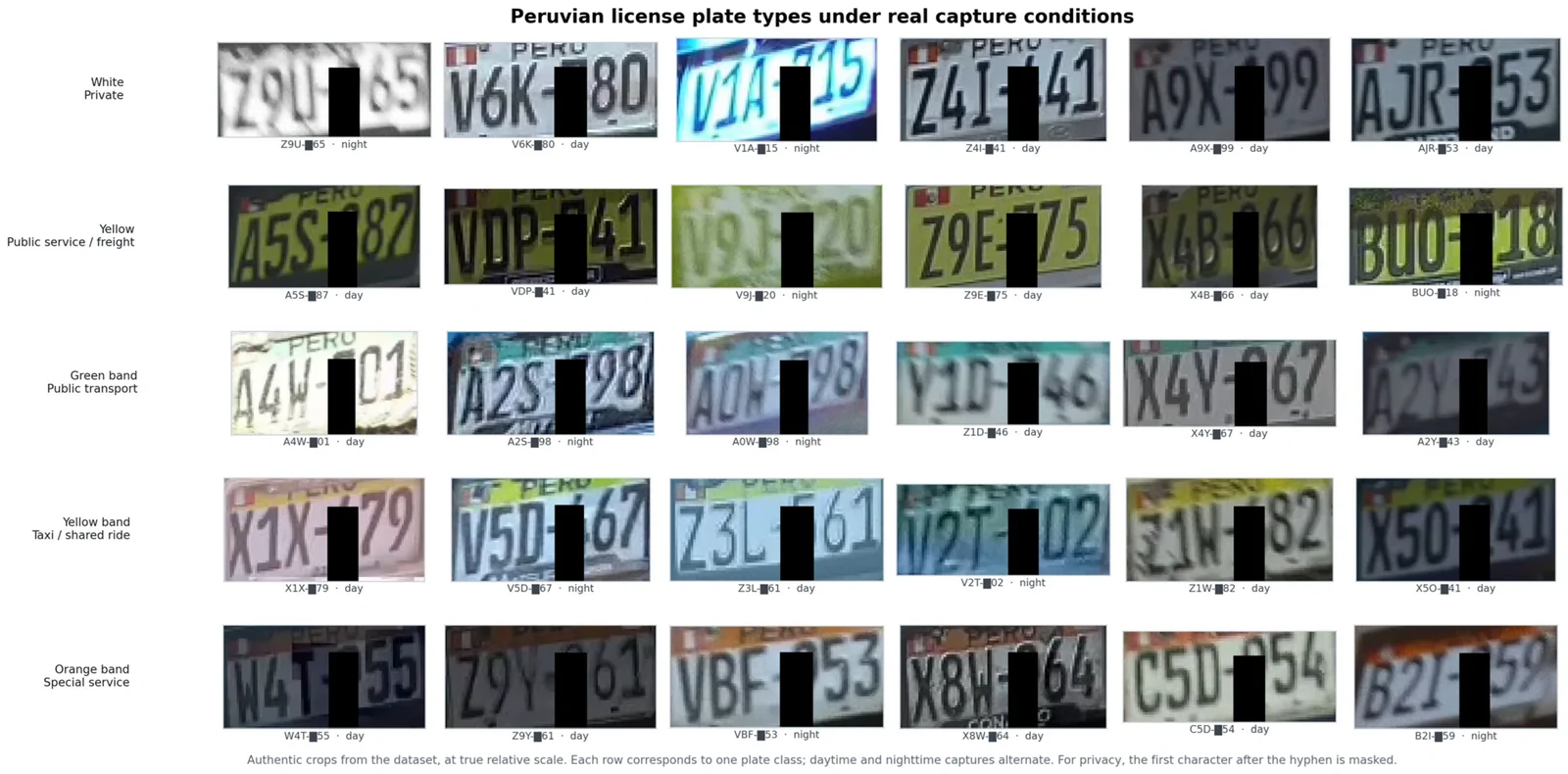
Peruvian plate types present in the dataset, under real capture conditions. Each row corresponds to a class defined by color and band under national regulations; daytime and nighttime captures alternate, and the crops are shown at true relative scale so that the disparity in resolution is visible. All share the six-character LNN-DDD pattern but differ in background, band, state of preservation and mounting.

Two properties of this population shape the design. The first is the skew of the frequency distribution: 52.8% of plates register one or two passes over the whole period, and only 7.9% exceed fifty. The other is the lighting distribution, which we compute from actual solar elevation at the site rather than from a clock cutoff: 96.38% day (elevation > 0°), 0.43% civil twilight (between −6° and 0°) and 3.19% night (≤−6°). Figure 2 shows the Peruvian plate types present in the dataset, under real capture conditions.

**Figure 3:**
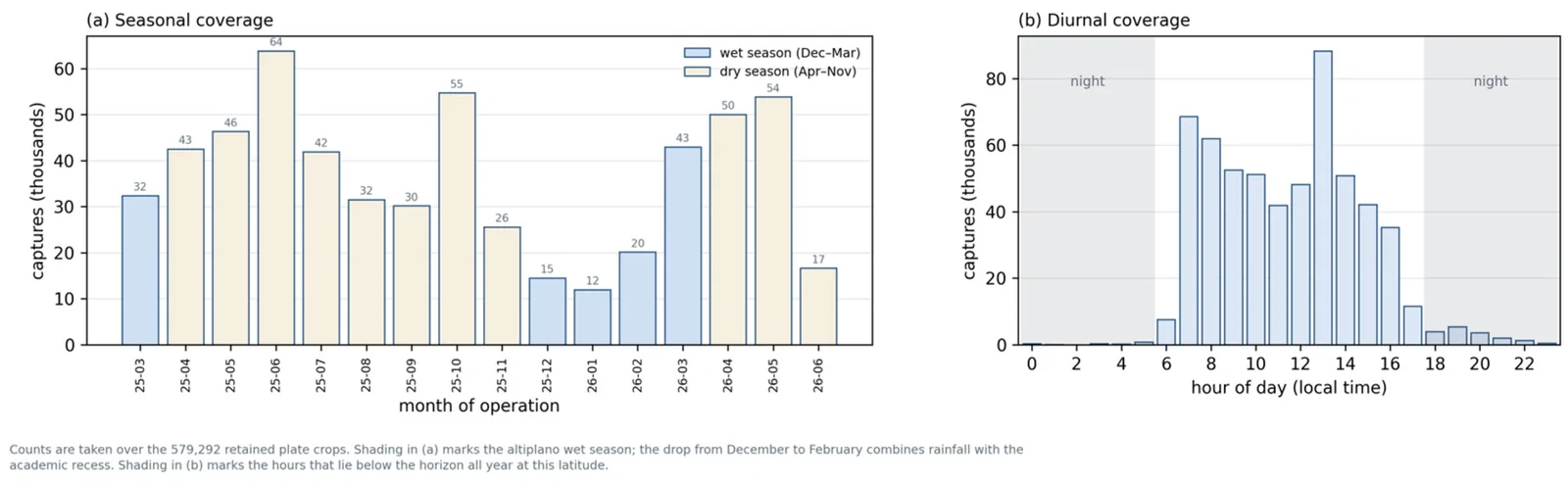
Seasonal and diurnal coverage of the corpus. (**a**) Captures per calendar month, with the altiplano wet season shaded. (**b**) Captures per hour of local time, with the hours that lie below the horizon all year shaded. The corpus covers one complete wet season and parts of two dry seasons, so seasonal variation in illumination, dust and precipitation is present in the training data rather than absent from it.

Figure 3 reports how those captures distribute across the calendar and across the day. The corpus spans sixteen calendar months and, therefore, covers one complete wet season and parts of two dry seasons. Volume falls from December to February, which conflates two causes that cannot be separated here: the rainy months and the academic recess. Within the day, the distribution is strongly bimodal, with peaks at the start of the working day and at midday and a long tail into the evening that supplies the nighttime captures.

Three features distinguish the Peruvian case from the settings that dominate the ALPR literature. The first is the plate taxonomy. Peruvian regulation encodes vehicle class in the background color and in a colored band, so a single national format spans white private plates, yellow plates for public service and freight, and three band variants for public transport, taxis and special services (Figure 2). A recognizer trained here must be invariant to five background appearances rather than one, which is a harder photometric problem than a single-color national stock.

The second is the character grammar. The national pattern LNN-DDD fixes three alphanumeric positions followed by three digits, which is the property that makes a deterministic positional corrector feasible; in jurisdictions with variable-length or multi-pattern plates, the same device would not apply. The third is environmental, and it is the one most often absent from public datasets: sustained operation above 3800 m, where radiation is intense, the thermal range within a day is wide, and the retroreflective surface of the plate interacts with nighttime infrared illumination in a way we were able to measure (Section 4.3.5). None of the public corpora we reviewed combines these three.

Both asymmetries imply that sampling by logged access and sampling by distinct plate yield different populations, which motivates the design of two complementary evaluation sets (Section 3.3.6).

#### 3.3.5. Partitioning

The guiding principle of the split is to group by entity rather than by file. Each plate contributes nineteen frames on average, so a random split by frame would place near-identical images of the same vehicle on both sides of the partition. The splitting unit is, therefore, the plate. Table 2 gives the resulting allocation.

We verified that no plate crosses partitions. The allocation deviates from the 80/10/10 planned over plates, which is to be expected since plates do not contribute equal numbers of frames; faced with the trade-off, the absence of leakage took precedence over exact balance.

To characterize transfer across optics, we incorporated a second domain: 2154 photographs of Peruvian plates taken with mobile devices and published under an open license. It had to be rebuilt before use. Its split was made by file after augmentation was applied, with the result that 73% of the source images in the test subset also appeared in the training subset. We reduced it to unique source images and re-split it, grouping by source.

#### 3.3.6. Ground Truths and Annotation Process

Recognition ground truth comes from checking the text of each plate against the official vehicle registry, the only source capable of resolving the ambiguities the image leaves open. We built two sets with deliberately different purposes, plus a control subset. Table 3 summarizes the role of each.

The unit of analysis deserves a precise statement, because crop and plate do not coincide entirely. Gold-B starts from one crop per plate, but 53 of its 1074 plates contribute more than one: these are cases in which the verifier retained several captures of the same plate so as not to discard divergent readings. The resulting 1130 rows constitute the unit over which all metrics and paired tests are computed. That 4.7% of non-independent rows is small, but we declare it because repeated-measures tests assume independence between units. Gold-A is different by construction: sampling by logged access, its 782 crops come from 524 plates and a habitual vehicle appears up to ten times, which is precisely the by-event view that set is meant to estimate.

The size of Gold-B was decided by a simulation-based power analysis. With the 126 clean plates available beforehand, power to detect a two-point difference was 0.07; at 900, it rises to 0.80. Sampling was stratified by lighting class and by plate-area quartile.

To estimate annotation reliability, two independent assessors read a blind subsample of 200 plates. They agreed with each other on 99.0% of plates (Cohen’s κ = 0.998), but against the official registry, each stopped at 92–93%. That gap marks the ceiling attainable from the image alone.

Detection ground truth consists of the 2600 images with manually annotated localization described above.

### 3.4. Phase 2: Character Recognition

#### 3.4.1. ROI Cropping and Normalization

The detector box is cropped from the original image at its native size, with a median of 101 × 51 px. That crop is the input to every system under comparison, so any observed difference is attributable to the recognizer and not to detection [18].

The general-purpose OCR engine works directly on it, with its own internal preprocessing. The compact recognizers cannot: they require a fixed-size input. For them, the crop is resized to 32 × 100 px in RGB, with values scaled to the [−1, 1] interval. Normalization, therefore, belongs to the student branch and not to the common chain, and its sole purpose is to feed LPRNet and CRNN identical inputs so that the two remain comparable.

The resolution was not chosen by convention but after an ablation. The median native crop has an aspect ratio of 1.98, with an interquartile range of 1.85 to 2.08: the Peruvian plate is very nearly 2:1, whereas the inputs customary in the literature distort it. Section 3.5.2 describes the experiment and Section ROI Normalization Resolution its outcome.

#### 3.4.2. Systems Compared and Experimental Objective

Six systems are compared; they constitute the levels of the main factor and are described in Table 4. The four trained ones cover both architectures under both supervision regimes, so the comparison between them can be made within each regime and not only in aggregate.

The two trained architectures answer to opposite design philosophies, and that is exactly what makes the comparison informative. LPRNet is purely convolutional: the height of the feature map collapses to one, and the width takes over as the temporal axis. CRNN adds two bidirectional recurrent layers over that axis, contributing context between neighboring positions at the cost of nine-times more parameters. Both are trained with CTC loss over 37 classes (36 alphanumerics plus the blank symbol) and decode in parallel, without autoregression and without segmenting characters: CTC produces a distribution per temporal position, and the reading follows from collapsing repetitions and blanks.

**Figure 4:**
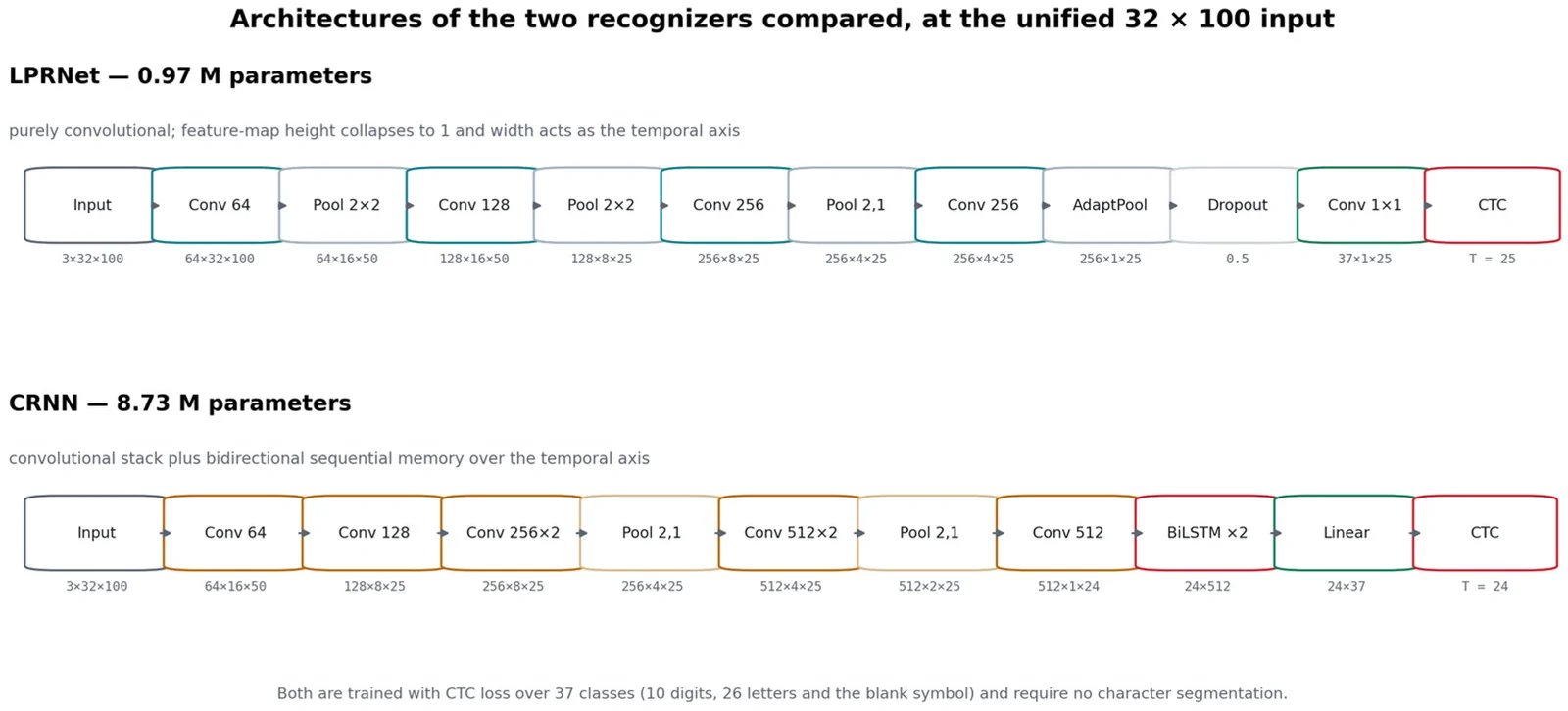
Architectures of the two recognizers compared, with the actual dimensions of each stage. LPRNet is purely convolutional: the height of the feature map collapses to one and the width acts as the temporal axis. CRNN adds two bidirectional recurrent layers over that axis. Both are trained with CTC loss over 37 classes and require no character segmentation.

The absence of segmentation is not a design preference but a consequence of the sensor. With a median plate of 101 × 51 px, each of the six characters has roughly 17 pixels of width, and those in the lower quartile fall below 14. At that scale, the boundaries between adjacent characters cannot be located reliably, so any pipeline that segments before recognizing propagates its cropping errors into the classifier. CTC loss avoids that step by aligning the output sequence with the horizontal axis of the feature map, without requiring a position-by-position correspondence. Figure 4 details the dimensions of each stage.

The experimental objective is twofold. We want to know under which evaluation conditions the claim holds that a compact distilled recognizer outperforms the procedure that generated its labels. And we want to know how the two architectures rank within each supervision regime, comparing them on the same input and with enough repetitions to separate a real difference from training variability.

#### 3.4.3. Distillation Learning

The compact recognizers are trained on the silver labels the teacher produces over the whole corpus. The scheme is a weakly supervised distillation [15,16], with one nuance relative to the usual formulation: here, the teacher is not a costly network being compressed but a generic OCR engine reinforced with domain rules. Nor does the student imitate an output distribution; it reproduces text strings. Transfer, therefore, occurs at the level of the label rather than of the logits.

We train on a random subsample of 100,000 crops drawn from the 459,598 in the training partition and use 10,000 from the validation partition to select the epoch that is kept. The subsample answers to the cost of the protocol: every configuration is repeated across several seeds and, in the resolution ablation, across several inputs. Adam optimizer is used, with learning rate 10^−3^ with cosine annealing, batches of 256 and 10 epochs.

#### 3.4.4. Fine-Tuning

The distilled model is followed by fine-tuning on verified ground truth. Plates are chosen by active selection: we retain those on which the available models disagreed with one another, which are the ones that contribute most information per unit of annotation effort. This yields 1850 crops from 1333 plates, with Adam, learning rate 3 × 10^−4^, batches of 64 and 25 epochs.

The purpose is not only to gain accuracy. It is to contradict the systematic biases of the teacher, which more silver data would merely repeat.

#### 3.4.5. Set Separation and Leakage Control

The protocol separates three disjoint roles, and the distinction is not cosmetic. Choosing the epoch that maximizes accuracy on the very set later reported amounts to selecting a model on the test set, and that produces an optimistic estimate. Table 5 sets out the allocation.

The held-out subset shares no plate with either the fine-tuning set or the selection set, and the procedure verifies this and logs it before training. The concrete hyperparameters of both stages are given in Section 4.2.

### 3.5. Experimental Analysis Phases

Beyond the processing flow, the methodology incorporates two analyses aimed at making the comparison between systems rigorous.

#### 3.5.1. Complexity Analysis

For each recognizer, we record three quantities: parameter count, size of the model exported to ONNX, and per-image latency measured on the same GPU with the same input. What decides which model ships on the edge server is not accuracy alone but its relation to cost. An accuracy difference is actionable only once its price is known.

#### 3.5.2. Controlled Ablation Study

The ablation isolates the effect of normalization resolution while holding everything else constant: same data, same protocol, same seed, same epochs. We compare five inputs, spanning 2256 to 12,800 px and aspect ratios from 2.00 to 3.92. Among them are the two default configurations of the original architectures and three that respect the native aspect ratio of the Peruvian plate. Each architecture–input combination is trained from scratch.

The design carries one constraint worth flagging. Both architectures embed a fixed downsampling stack, computed so that the height of the feature map collapses to one at their nominal resolution. Admitting other inputs forces that collapse to be replaced by an adaptive mechanism, which makes the network runnable but does not scale its depth. As the image grows, the receptive field covers a smaller fraction of each character. The ablation, therefore, confounds aspect ratio with effective receptive field in shallow architectures, and its results must be read with that caveat, which we detail in Section ROI Normalization Resolution.

## 4. Experiments

### 4.1. Dataset and Experimental Configuration

Before presenting the results, we specify what they are measured on: the sets used, the execution environment and the metrics by which the system is judged.

#### 4.1.1. Data Used in Each Experiment

The corpus, its filters and its allocation were described in Section 3.3.4 and Section 3.3.5. What matters here is which portion takes part in each experiment, which is considerably smaller than the total and should not be confused with it.

No model is trained on the 579,292 frames. Distillation runs over the random subsample of 100,000 silver crops drawn from the training partition (Section 3.4.3), and the epoch that is kept is decided on 10,000 validation crops. The subsequent fine-tuning uses 1850 verified crops from 1333 plates. The two PaddleOCR-based systems are not trained at all: they are the teacher, and they are measured exactly as they were used to label.

The reported figures come from the three ground truths in Table 3. The main evaluation is Gold-B, with 1130 crops. Gold-A, with 782, estimates accuracy per logged access and additionally decides the fine-tuning epoch, roles that Table 5 keeps separate. The held-out subset of 689 crops acts as a leakage control and takes part in no gradient and no selection.

#### 4.1.2. Hardware and Software Environment

Everything ran on an NVIDIA RTX 5060 Ti GPU with PyTorch 2.13 and CUDA. Detection uses Ultralytics 8.4; the statistical analysis, SciPy 1.18 and statsmodels 0.14 on Python 3.12. Deployed models are exported to ONNX, and we verify that format by contrasting its predictions with those of the original checkpoint, plate by plate.

The code for dataset generation, training, evaluation, testing and figures is openly available, and every figure appearing here is regenerated by a single procedure that recomputes the master table over the three evaluation sets. The plate crops are deposited separately, on Zenodo and under a use agreement, because they constitute personal data (see Data Availability Statement).

#### 4.1.3. Evaluation Metrics

The primary metric is exact match: the proportion of plates whose complete reading coincides with the official registry, with no tolerance for partial errors. It is the one that matters in operation, because a reading with a single wrong character no longer identifies the vehicle. As a secondary metric, we report character error rate (CER), which conveys the severity of failures, and as a diagnostic, the proportion of readings whose length departs from the six characters of the national format.

For detection, we use mAP@50, mAP@50–95, precision and recall. We add a per-image binary metric that asks whether all plates in an image were localized with an intersection over union (IoU) of at least 0.5, which allows paired tests between detectors.

#### 4.1.4. Statistical Analysis

Main test. H_0_: the k systems have the same success proportion; H_1_: at least one differs. Since the response is dichotomous and the k systems are evaluated on the same plates, that is, with repeated measures, the omnibus test used is Cochran’s Q, which generalizes McNemar’s test to more than two related treatments. A one-way ANOVA would be inadequate in this design: it presupposes a continuous response, normality of residuals, homoscedasticity and independence of observations, and none of the four assumptions holds.

Post hoc tests. The k(k − 1)/2 pairwise comparisons use the exact binomial version of McNemar’s test, valid when the number of discordant pairs is small, and are corrected by Holm–Bonferroni, uniformly more powerful than Bonferroni and valid without assuming independence between tests.

Intervals and effect sizes. Each proportion carries its Wilson interval; each paired difference, a paired bootstrap interval with 10,000 replicates, resampling plates rather than predictions so that correlation is preserved, plus Cohen’s g for paired data. Comparisons across disjoint subgroups (lighting classes, frequency bands, optical domains) are unpaired and use Newcombe’s method for the difference of two independent proportions.

Factorial model. We fit a binomial generalized linear model with a logit link over the system × lighting class × frequency design, with robust errors clustered by plate, since each plate contributes six correlated observations and omitting the clustering would understate the standard errors. Terms are tested by Wald statistics.

Comparison between architectures. The tests above compare concrete models over a population. Comparing architectures requires incorporating training variability, so those comparisons are made across runs: each architecture is trained with three seeds, and the test is a Welch t on the means, which does not presuppose equal variances. With three repetitions, the test has little power, so a non-significant difference here should be read as absence of evidence and not as evidence of equivalence.

A significance level of α = 0.05 is adopted throughout.

### 4.2. Training and Inference Details for the OCR Models

The two recognizers are trained by the same procedure, so that any difference between them is attributable to the architecture. Table 6 gives the configuration.

Preprocessing. Each crop is resized to 32 × 100 px in RGB and its values scaled to [−1, 1]. During distillation, a random brightness and contrast perturbation is applied to 30% of the samples; no augmentation is applied at validation or inference.

Epoch selection. In both stages, we keep the checkpoint that maximizes exact match on the selection set, never on the set later reported. This safeguard proved necessary: validation accuracy peaks between epochs two and seven and declines thereafter, while the training loss keeps falling to 0.001, an overfit to the silver labels that costs between five and ten points if the last epoch is taken instead of the best one.

Repetitions. Each configuration is trained with three seeds and reported as the mean with its standard deviation, which is 0.84 points for LPRNet and 1.51 for CRNN. That value sets the threshold below which a difference between two individual runs is not interpretable, regardless of what a paired test on one of them may indicate.

Inference. Decoding is greedy CTC, without a language model, without format constraints, and without the deterministic corrector: the distilled recognizer operates on its own. Latency is measured on the same GPU, with unit batch size and after ten warm-up passes. Deployed models are exported to ONNX and their predictions contrasted plate by plate with those of the original checkpoint.

### 4.3. License Plate Recognition Results

#### 4.3.1. Recognition on the Two Ground Truths

Table 7 contains the central result of this work. The same six systems are evaluated on the two ground truths, and the orderings and magnitudes they produce differ substantially.

The teacher shows by far the largest variation between the two views. PaddleOCR with corrector reaches 95.40% under the by-event view and collapses to 54.69% under the by-plate view: 40.7 points between two figures from the same system. Fine-tuned LPRNet, by contrast, gives up only 5.5 points between one view and the other, and fine-tuned CRNN 7.5. We identify the cause in Section 4.3.4.

The ordering of the two architectures is also informative, since it holds across all four blocks: LPRNet ahead of CRNN under silver labels and equally so after fine-tuning, on both ground truths, with nine-times fewer parameters. Section 4.3.8 tests that difference with training variability folded in.

#### 4.3.2. Global Comparison Between Systems

Omnibus. Cochran’s Q over the 1130 crops of Gold-B gives Q = 1389.43 (df = 5), with 628 informative crops, that is, those on which not all systems agree. The hypothesis of equal performance is rejected.

**Figure 5:**
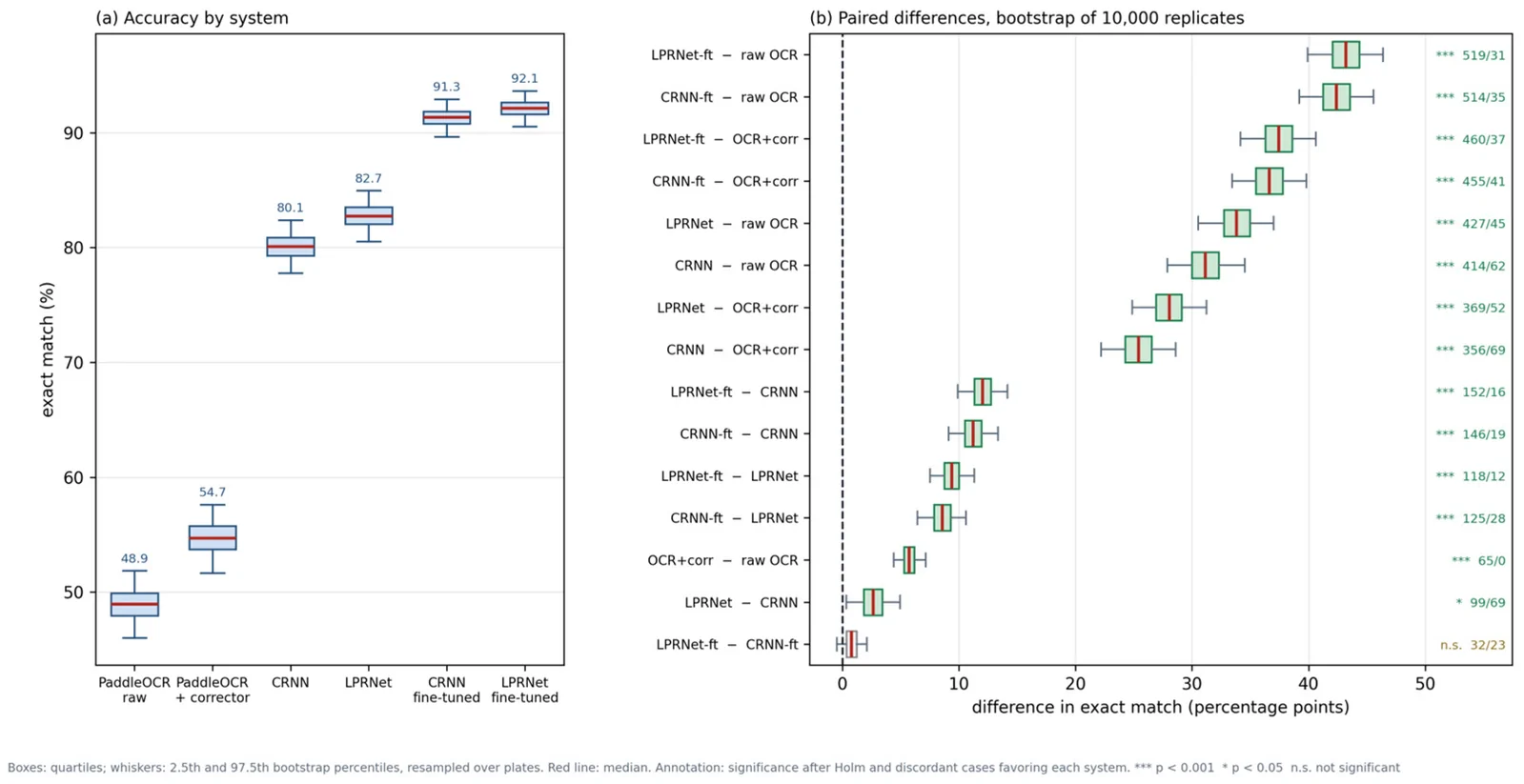
Post hoc tests on Gold-B by paired bootstrap with 10,000 replicates, resampling plates to preserve the correlation between systems. (**a**) Distribution of the exact match of each system. (**b**) The fifteen paired differences, ordered by median, with the zero line marked. Boxes span the quartiles and whiskers the 2.5th and 97.5th percentiles; the red line is the median. In the margin, significance after Holm correction and the number of discordant plates favoring each system. Green boxes are the significant comparisons; the two gray ones at the bottom, which pit architectures against each other within the same supervision regime, are the only ones that are not.

Post hoc. Of the fifteen pairwise comparisons, thirteen are significant after Holm–Bonferroni correction. The two that are not pit models of the same family against each other: fine-tuned LPRNet against fine-tuned CRNN stands at *p* = 0.281, and their silver counterparts fall at the threshold, *p* = 0.050. Table 8 details the tests, and Figure 5 displays them with their bootstrap distribution.

The count of discordant pairs qualifies what the p value suggests. The corrector against raw OCR shows the most regular pattern in the table: of the 65 discordant cases, all 65 favor the corrector and none the uncorrected engine, so the corrector never worsens a reading; it only fixes it or leaves it unchanged. At the opposite end, fine-tuned LPRNet against fine-tuned CRNN splits 32 to 23, a difference that the Holm correction does not declare significant.

#### 4.3.3. Factorial Model

The binomial generalized linear model over the system × lighting × frequency design, with errors clustered by plate, finds all three main effects and one interaction significant, as Table 9 records.

**Figure 6:**
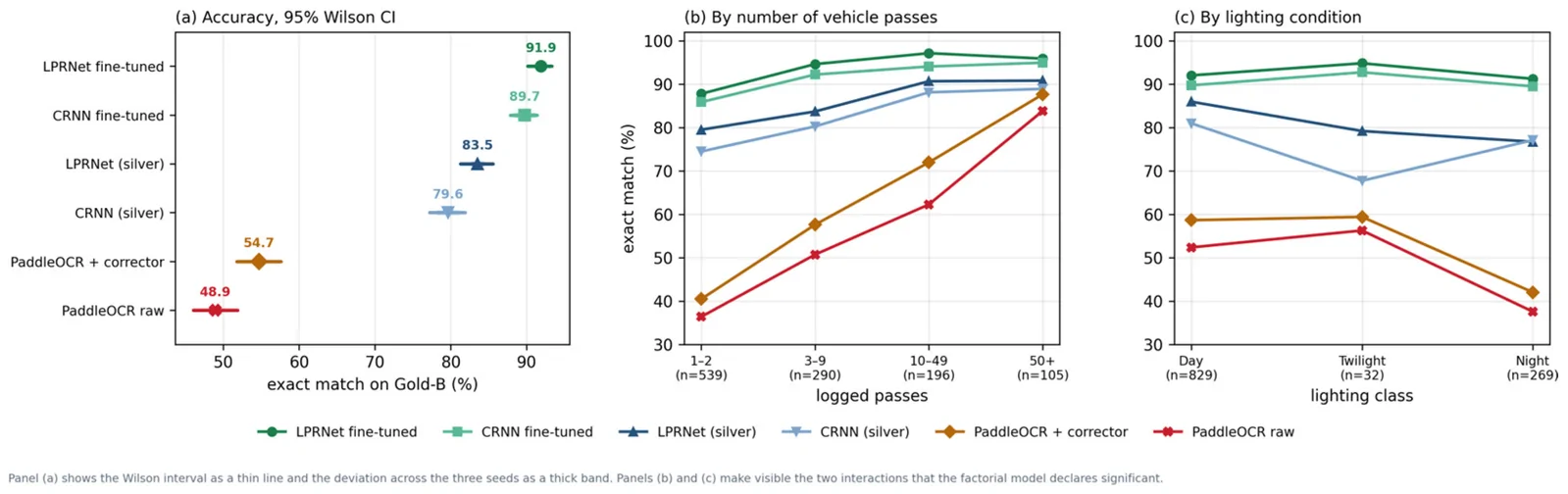
Comparison of the six systems on Gold-B. (**a**) Exact match with its 95% Wilson interval as a thin line and the deviation across the three seeds as a thick band. (**b**) Profile by number of vehicle passes and (**c**) by lighting condition: these are the two interactions the factorial model declares significant. The steep slope of the two PaddleOCR systems in (**b**) is the frequency dependency the distilled models do not inherit; their drop in (**c**) on moving to night is the one that fine-tuning cancels.

What dominates are the interactions, not the main effects. Both come out clearly significant: the effect of the system depends on how many times the vehicle passed and also on the lighting condition. Figure 6b,c render them as profiles, which is where one sees at a glance what the table merely states.

The main effect of lighting is not significant (*p* = 0.483), a result that calls for interpretation rather than dismissal. Averaged over the six systems, day and night are hardly distinguishable, because the two fine-tuned models are nearly immune to illumination and offset in the mean what the others lose. But that insensitivity is precisely what the interaction measures: night costs the corrected OCR 16.6 points, between 3.8 and 9.2 to the silver models, and less than a point to the fine-tuned ones. A small main effect alongside a large interaction does not mean lighting is irrelevant; it means its effect depends entirely on which system faces it.

Note also that in the presence of interactions, the main effects are conditional on the reference levels; what answers globally whether the system matters is Cochran’s Q.

#### 4.3.4. Effect of Visit Frequency: Single-Pass Versus Multi-Pass Plates

The consensus mechanism described in Section 3.3.3 can only help a plate that was seen more than once, so the question is how much accuracy the system loses on the majority of plates that it cannot help. Table 10 stratifies by the number of retained captures per plate, which is the observable proxy for visit frequency; the four bands correspond to roughly one, two to five, five to twenty-five, and more than twenty-five actual passes, since deduplication keeps a mean of 2.0 crops per pass. The stratification explains the teacher’s collapse between the two views.

The corrected OCR goes from 40.4% on plates with one or two retained captures to 87.6% on those with fifty or more. That is 47.2 points of range on the same system. Fine-tuned LPRNet covers 8.1 points between the same extremes and fine-tuned CRNN 9.1. The reason lies in the capture: the system consolidates each plate’s label by consensus across passes (Section 3.3.3), so the reliability of the weak supervision grows with the vehicle’s frequency. The distilled models do not inherit that dependency because, during training, they average out labeling noise instead of reproducing it case by case. The gradient does not vanish entirely in them, but it drops from being the dominant factor to a single-digit variation. This is the practical consequence for deployment: on the 63.2% of plates that the consensus cannot assist, the teacher is not a usable recognizer, while the distilled student remains within nine points of its own best case.

#### 4.3.5. Robustness to Plate Size

The size analysis is carried out on Gold-A and not on Gold-B. The latter deliberately oversamples night, 24% against 3.2% in the corpus, and area turns out to correlate with the lighting condition: its quartile of large plates contains 34.0% nighttime captures against 11.2% in the small quartile, most likely because the retroreflective plate saturates under infrared illumination and the detector draws a larger box. Stratifying Gold-B by area would, therefore, measure illumination to a substantial degree.

Table 11 makes a sharp pattern visible. The uncorrected OCR engine is the only system that collapses on small plates: it falls to 45.1% in the bottom quartile against 73.5% in the third, nearly thirty points apart. The corrector raises it to 94.3% in that same quartile, and the compact recognizers reach 92.4% there without correction and 98.1% after fine-tuning, with no post-processing whatsoever. What transfers to the student is, precisely, the teacher’s robustness in the hard case.

The top quartile gives up a few points across all systems, the fine-tuned ones included. With the camera at 2.50 m and an angle below 45°, plates of larger apparent area belong to the closest vehicles and are, therefore, captured with the strongest perspective distortion. The drop is small and will not support a formal test, but it suggests that the difficult regime of this installation has two ends rather than one.

#### 4.3.6. Robustness to Lighting Condition

The nighttime oversampling of Gold-B allows a regime that the full corpus barely represents to be characterized. Table 12 breaks accuracy down by lighting class.

Between day and night, the corrected generic OCR loses 16.6 points. Fine-tuned LPRNet gives up 0.8 and fine-tuned CRNN 0.2. Distillation does not merely compress the model; it stabilizes it against a condition the teacher cannot handle. Note that the stability comes from fine-tuning rather than from distillation on its own, since the silver models are affected by nighttime capture, with drops of 9.2 and 3.8 points. The twilight subset is small (*n* = 32), and its values should be read as indicative; that fine-tuned LPRNet obtains its best figure there is sampling noise, not an effect.

#### 4.3.7. Effect of Fine-Tuning and Leakage Control

Fine-tuning on actively selected verified ground truth improves the recognizer on both sets, the held-out subset included, in which no plate takes part in the gradient or in the epoch choice. Table 13 reports both figures.

The gain is confirmed on plates foreign to the entire procedure, which rules out memorization: LPRNet rises 2.95 points on the held-out subset and CRNN 4.36, in both cases several-times the deviation across seeds. On the held-out subset, the magnitude is smaller than on Gold-B, and there is an explanation: only 10.3% of its plates carried an erroneous automatic label, against 45.3% of the population, and it is precisely in those hard cases that fine-tuning contributes most.

Note that CRNN gains more than LPRNet from fine-tuning, 10.08 points against 8.35, and still does not catch up. It recovers part of the initial gap under clean supervision, but not all of it, which fits the interpretation that its additional capacity goes into fitting the noise of the silver labels rather than into learning the typographic regularity.

#### 4.3.8. Training Variability and Comparison Between Architectures

Each architecture was trained with three seeds under both regimes, always evaluating on the same external set. Table 14 records the dispersion obtained.

Seed-to-seed variability is not negligible and differs between architectures: 0.57 to 0.84 points in LPRNet against 1.48 to 1.51 in CRNN. That is the threshold below which a difference between two individual runs is not interpretable, and it explains why a single evaluation can mislead.

The ordering between architectures does not change with the supervision regime. LPRNet leads in both: +3.89 points under silver labels (t = 3.90, df = 3.1, significant at 5%) and +2.15 points after fine-tuning (t = 2.35, df = 2.6, not significant). The correct reading of that second test is that with three repetitions, the test has little power, so there is absence of evidence of a difference rather than evidence of equivalence. What can be asserted is that CRNN, with nine-times more parameters, outperforms LPRNet in neither regime.

This result corrects an earlier conclusion of our own work. A previous cross-validation had found CRNN superior to LPRNet after fine-tuning, which suggested that the ordering inverted with the supervision regime. That comparison, however, pitted LPRNet at 24 × 94 px against CRNN at 32 × 100 px, their respective nominal resolutions. Once the input is unified, the inversion disappears. This case reproduces the same phenomenon documented along the sampling axis: a protocol detail, apparently innocuous, is enough to reverse the conclusion about which architecture is better.

As for the accuracy–size trade-off, fine-tuned LPRNet is both the most accurate and the most readily deployable, at 3.7 MB in ONNX against some 35 MB for CRNN. No accuracy–cost trade-off arises in this case.

### 4.4. Ablation Results

#### ROI Normalization Resolution

Table 15 records what effect the normalization resolution has on exact match in Gold-B.

On CRNN, which is the interpretable arm, respecting the native aspect ratio contributes no accuracy: the four comparisons against its nominal configuration fall between +0.54 and +1.24 points, and none reaches significance after Holm correction. Nor does the prediction about reading length hold: the 48 × 96 input halves the proportion of outputs with length other than six (1.15% against 2.12%, *p* = 0.052), but 64 × 128 and 80 × 160 share the same aspect ratio and do not improve (2.04% and 1.77%). If the ratio were the cause, all three should move in the same direction. Enlarging the input does increase latency monotonically.

The LPRNet rows at 2:1 do not measure what they were meant to. Their training loss stalls at 0.18–0.45 against 0.011 for the nominal configuration, that is, the model does not converge. The cause is the one flagged in Section 3.5.2: enlarging the input without scaling the depth of a network of only four convolutional blocks leaves the receptive field covering a fraction of a character. CRNN is less affected because it downsamples more and because its recurrent layers aggregate context along the width. The experiment is, therefore, confounded for shallow architectures and is reported as a negative result, not as evidence that aspect ratio is irrelevant in general.

We adopt 32 × 100 for three reasons. It is the input at which LPRNet obtains its best figure. It leaves CRNN 1.24 points below its own best, less than its seed-to-seed deviation and without significance after Holm, so no appreciable penalty is imposed on it. And it is CRNN’s nominal resolution, close to LPRNet’s, which keeps both networks well clear of the regime in which the shallower one stops converging. Unifying it also allows both to be fed identical tensors, a requirement if the comparison between them is to be attributable to the architecture.

### 4.5. Error Analysis in License Plate Recognition

#### 4.5.1. Qualitative Error Analysis

**Figure 7:**
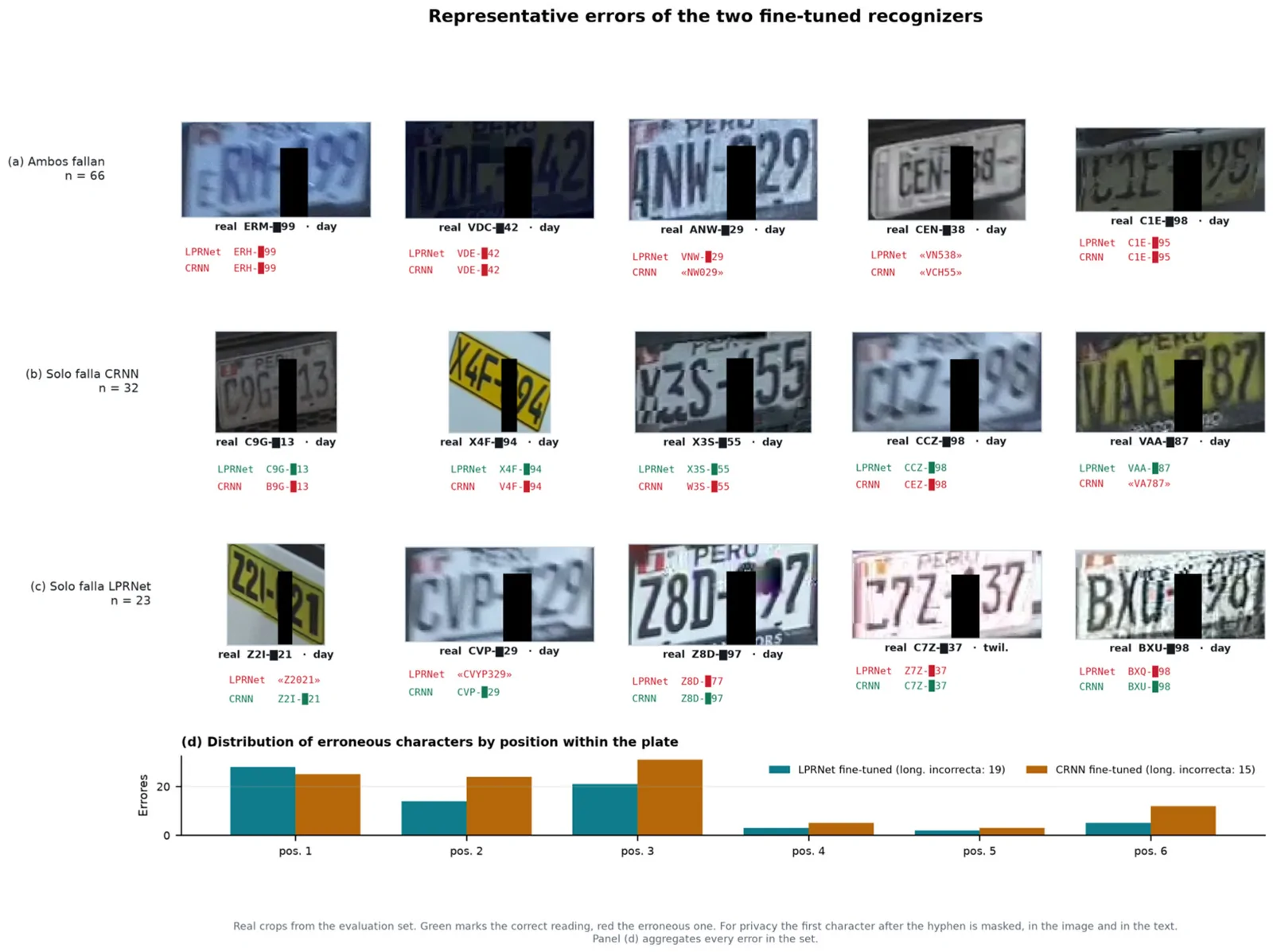
Representative errors of the two fine-tuned recognizers on Gold-B. (**a**) Both fail: intrinsically difficult plates that mark the ceiling of the method. (**b**,**c**) Cases that only one of the two resolves. Green marks the correct reading and red the erroneous one; strings in angle quotes are outputs of length other than six. The first character after the hyphen is masked, in the image as well as in the text; the examples were chosen so that the error shown does not coincide with that position. (**d**) Distribution of erroneous characters by position over the whole set.

Figure 7 groups the failures of the two fine-tuned recognizers into three categories. On Gold-B, evaluated with the reference seed so that the counts match the paired tests of Table 8, 66 plates are read incorrectly by both models, 32 by the CRNN alone and 23 by LPRNet alone. The two models, therefore, fail together on the large majority of their errors, and the plates each one resolves on its own are few enough that the difference between them rests on a small number of cases.

Errors in both models concentrate in the three-character prefix. Of the 73 positional errors LPRNet makes on readings of correct length, 63 fall in positions 1 to 3 and only 10 in positions 4 to 6; for the CRNN, the split is 80 against 20 out of 100. That is 86% and 80% of positional errors confined to the first half of the string. The asymmetry follows from the format: the first three positions admit both letters and digits, whereas the last three are always digits, which shrinks the confusion space to ten classes and removes the O/0 and I/1 ambiguity entirely.

The two architectures also differ in how they fail, although less sharply than the parameter gap might suggest. LPRNet returns a reading whose length departs from six characters in 19 of 1130 cases (1.7%) against 15 (1.3%) for the CRNN. The direction is the one predicted by the architectures: greedy CTC decoding can merge adjacent activations when characters are close together or poorly contrasted, and the recurrent layers of the CRNN carry sequential context that makes that collapse less likely. The magnitude, however, does not support a strong claim, and it does not propagate to the outcome that matters. LPRNet fails alone on 23 plates against the CRNN’s 32, so whatever the recurrent layers gain on string length, they lose elsewhere, and the global ordering of Section 4.3.8 is unaffected.

#### 4.5.2. Inter-Annotator Agreement and the Human Ceiling

Two independent assessors blindly read 200 plates. Agreement between them is nearly perfect (99.0% per plate; Cohen’s κ = 0.998), and their only two discrepancies correspond to the O/0 confusion, the same one the corrector addresses. Against the official registry, each assessor reaches 92.0% and 93.0%: even expert readers who agree with one another diverge from the registry on intrinsically ambiguous plates. Table 16 collects the three comparisons.

This exercise was carried out on Gold-A, so the 92–93% human figure is comparable with the 97.36% of the fine-tuned recognizer under that same view and not with its 91.89% on Gold-B. The human ceiling over arbitrary plates has not been measured and would predictably be lower.

### 4.6. Detection Subsystem Results

#### 4.6.1. Comparison of Detection Architectures

On the entity-grouped split, the ten trained architectures still reach mAP@50 between 0.9947 and 0.9950 and recall between 0.9974 and 1.0000, so those two metrics are saturated and cannot discriminate. The differentiating factor is mAP@50–95, which measures box tightness. Table 17 reports all ten under the clean split alongside the figure each obtained under the original random split.

Removing the leakage costs between 0.47 and 3.05 points of mAP@50–95, 1.39 on average, while leaving mAP@50 and recall essentially untouched. The inflation was, therefore, real but bounded, and it concentrated in box tightness rather than in whether the plate was found at all, which is consistent with near-duplicate images easing localization refinement more than detection itself. Two architectures lose most: RT-DETR-l at 3.05 points and YOLOv12m at 2.78, both of them among the larger models, which is what one would expect if extra capacity were partly memorizing repeated scenes.

The ranking does not survive intact. Kendall’s tau between the two orderings is 0.64, with 8 of the 45 pairs inverting, although the extremes hold: YOLOv8s remains the most accurate and RT-DETR-l the least. This is a third instance of the phenomenon this study documents along the sampling and the preprocessing axes. A partition detail that looks like bookkeeping reorders the middle of an architecture comparison, and a study that reported only the leaky ranking would have recommended YOLOv8m and YOLOv12m above models that actually beat them.

The deployment choice survives the correction, though less comfortably than before. Among the nano variants, the clean split gives YOLOv12n 0.9258, YOLOv8n 0.9201 and YOLOv11n 0.9192, a spread of 0.66 points, against latencies of 1.30, 1.18 and 1.27 ms. YOLOv8n is the fastest and sits 0.57 points below the best nano, so it remains a defensible choice for this installation, but the original table overstated the case: what looked like a clear call is a near-tie, and a redeployment today would reasonably prefer YOLOv12n for 0.12 ms more per image. The small variants no longer uniformly match or beat the medium ones either; with 1820 training images, the relation between capacity and accuracy is weak in both directions.

#### 4.6.2. Cross-Optics Gap of the Detection Subsystem

The preceding figures are in-domain: they come from the same camera and installation. Evaluated on the 323 photographs that form the test partition of the second domain, the 2154-image mobile-device set rebuilt in Section 3.3.5, the same detector behaves very differently, as Table 18 shows.

The detector loses six plates out of every ten when the optics change. A detector retrained on the union of both domains, subsampling the dominant one so that it does not saturate the set, raises recall to 0.874 and mAP@50 to 0.911 without losing performance on the original camera (mAP@50–95 of 0.915 against 0.908). On the per-image binary metric, the paired test is conclusive: the merged detector correctly localizes all plates in 285 of 323 images (88.2%) against 128 (39.6%) for the original, with 158 images gained against one lost (exact McNemar, *p* = 4 × 10^−46^).

## 5. Discussion

### 5.1. The Corrector Enables Weak Supervision Without Being a Competitive Recognizer

Seen by event, deterministic correction looks impressive: recognition rises from 65.1% to 95.4%, thirty points, which invites the conclusion that nearly all the error of the generic OCR engine is disambiguation rather than reading. The by-plate view does not support that impression. There, the same corrector contributes 5.75 points, from 48.9% to 54.7%. The explanation is simple: positional rules fix the O/0 and I/1 ambiguity when the rest of the string is already right, but they do not recover a character that was genuinely misread, which is exactly what abounds among infrequent and poorly photographed plates. The corrector is, therefore, a cheap, training-free label generator, not a recognition system in its own right. The distinction is not academic: much of the literature evaluates correctors and recognizers with the same metric and on the same kind of set, without separating the two roles.

### 5.2. Distillation Produces Specialists That Outperform Their Teacher

On arbitrary plates, the fine-tuned student leads the teacher by 37.2 points. Two mechanisms account for it. The first is averaging: trained over hundreds of thousands of noisy labels, the model learns the underlying typographic regularity instead of the teacher’s case-by-case decisions and, for that reason, does not inherit its dependence on pass frequency. The second is fine-tuning, which contradicts the systematic biases of the OCR engine rather than repeating them, and repeating them is all that more silver data would achieve. The dominant error, the first letter of the regional series read as a digit and turned by the corrector into a different letter, disappears entirely. None of this is paradoxical. A student can surpass its teacher because the teacher is not an oracle but a procedure with structured errors.

### 5.3. Additional Capacity Does Not Convert into Accuracy

With nine-times more parameters, CRNN does not outperform LPRNet under either supervision regime. Under silver labels, it is 3.89 points below, a difference the across-seed test declares significant; after fine-tuning, the distance narrows to 2.15 points and ceases to be significant, although the sign does not change. A conservative interpretation is that the recurrent architecture offers no demonstrable advantage here, not that the two are equivalent; with three repetitions, the test has little power.

That sequential capacity does not pay off has a plausible explanation in the input regime. With six characters over 25 timesteps and a rigid national pattern, the context between neighboring positions that the recurrent layers contribute is information LPRNet already recovers through convolution. CRNN pays for that context with parameters that, on a set of 100,000 crops and with noisy labels, go into fitting noise. This is consistent with its greater seed-to-seed variability, almost double that of LPRNet under both regimes.

An earlier version of this work concluded the opposite, that the ordering inverted after fine-tuning, on the basis of a cross-validation in which each architecture ran at its nominal resolution. Once the input is equalized, the inversion disappears. This is reported because it is the same mechanism this study documents for evaluation-set sampling: a protocol detail that looks secondary decides the direction of the conclusion. This qualifies comparative architecture studies conducted without controlling the input [18], and argues for reporting the normalization resolution alongside the comparison, just as the dataset is reported.

### 5.4. One Run Is Not Enough to Compare Architectures

Over a single run, the two fine-tuned recognizers split 32 discordant plates against 23, and their difference does not reach significance (*p* = 0.281 after Holm). With training repeated across three seeds, the mean difference is 2.15 points in favor of LPRNet and again falls short (t = 2.35, df = 2.6). The two tests do not answer the same question: the first compares these two concrete models on this population, and the second compares the architectures with training variability folded in. Deciding which architecture is better requires the second, and answering it requires training more than once. That neither test declares significance, with CRNN carrying nine-times more parameters, is itself the actionable result.

### 5.5. Human Ceiling and Ground-Truth Validity

Two expert assessors agree with each other at 99% and with the official registry at only 92–93%. That gap delimits the ceiling attainable from the image alone and, incidentally, validates the use of the registry as ground truth. The comparison belongs where it was made: the exercise was carried out on Gold-A, so the 92–93% human figure compares with the recognizer’s 97.36% under that same view, not with its 91.89% on Gold-B. Over arbitrary plates, we have not measured the human ceiling, which would predictably be lower.

### 5.6. Implications for Deployment of the Sensing System

Three design decisions are justified by the evidence. Processing at the edge, to begin with, means the following: only the structured record leaves the local network, so the privacy problem is resolved at the source rather than mitigated afterwards. Shipping the compact recognizer, next, no concession is required: fine-tuned LPRNet is at once the most accurate and the smallest, 3.7 MB against 35 MB for CRNN, which removes the usual accuracy–cost trade-off at the edge or on a mobile device [9,10]. And the third, less obvious, is as follows: do not reuse the fixed-installation detector on other optics. Its 17% retention argues against it, and the merged dataset offers an alternative that has already been measured, recovering recall to 0.874 without degrading performance at the original installation.

### 5.7. Deployment Architecture and the Edge Bottleneck

The current topology streams 1280 × 720 frames at 10 fps over RTSP to an edge server on the same wired segment, where detection, cropping and recognition run in sequence. Detection itself is not the constraint: at 1.1 ms per image, a single YOLOv8n instance has roughly two orders of magnitude of headroom over a 10 fps stream. The constraint is the continuous transport of full frames, which occupies the link whether or not a vehicle is present and which scales linearly if further lanes are added.

That topology also concentrates the risk of frame loss at the worst moment. Deduplication retains a median of one crop per vehicle pass, so temporal redundancy is thin: a frame dropped during a burst of arrivals is not generally recoverable from a neighboring frame of the same pass. Only 28.0% of passes leave two or more retained crops. We measured no packet loss on the dedicated segment during the 465 days of operation, but the margin is structural rather than demonstrated, and it would narrow immediately on a shared or wireless link.

Running the detector on the camera itself, or on a low-power accelerator attached to it, would address both points at once. Only the cropped plate region would cross the network, which reduces the transported payload by roughly two orders of magnitude relative to the full frame, removes the dependence of recognition on uninterrupted transport, and tightens the privacy argument further, since full frames would never leave the camera at all. The compact recognizer already fits that budget: 0.97 M parameters and 3.7 MB in ONNX are within reach of current on-camera accelerators. We did not test this configuration because the installation was already in service when the study began and the camera in place exposes no programmable inference stage; it is the first item of the deployment work that follows from this study.

### 5.8. What Generalizes Beyond the Peruvian Case

A single-site study raises the question of which findings transfer. Three layers can be distinguished. The methodological findings are the most portable, because neither depends on the plate stock: that the sampling unit of the evaluation set can move a reported margin by an order of magnitude, and that the normalization resolution can reverse an architectural ranking, are statements about evaluation protocol and would hold for any ALPR system whose labels are consolidated across repeated observations of the same entity. Any access-control installation that deduplicates by plate accumulates exactly that structure.

The teacher–student recipe is portable under one condition: that the jurisdiction has a fixed positional grammar for its plates. Most of Latin America and much of the European Union satisfy it, and for those, the corrector can be reused with minimal modification. Where plate length varies, or where several patterns coexist without a reliable way to tell them apart from the image, the deterministic corrector is no longer applicable and a probabilistic decoder constrained by a pattern lexicon would be the natural substitute.

What does not transfer is the calibration. The 37.2-point margin, the 17% cross-optics retention and the quartile breakdown by plate area are properties of this camera, this mounting geometry and this altitude, and they are not expected to reproduce elsewhere. We report them so that another installation has a reference point for its own measurement, not as constants of the problem.

## 6. Limitations

Seven limitations bound the scope of the preceding results.

The first is that grouping by label is not the same as grouping by vehicle. The partitions were built by plate, but the plate used was the silver label, and that label is noisy: the same vehicle can receive different strings on different passes and end up on both sides of the split. On verifying Gold-B against the official registry, 396 of its 1130 crops, 35%, turned out to have a true label present in training, and all of them were plates the annotator had corrected. A frequency-controlled test places the residual effect at some 7 points over the affected subset, and a reweighted estimate that neutralizes it gives 89.1% instead of 91.9%. We report the complete figure as the primary one and the reweighted figure as a sensitivity analysis, because these are different frames, taken at another time, with another size and another illumination, in an uncontrolled environment.

The second is that the held-out subset supports a narrower conclusion than it appears to. It does rule out that the fine-tuning gain comes from memorization, but it cannot support a comparison between student and teacher. The reason lies in its exclusion criterion, which is not independent of the teacher: a plate is corrected precisely when its automatic label, which is the teacher’s output, is wrong. Discarding the corrected plates that coincide with training systematically removes the teacher’s failures. The consequence shows in the figures: the corrected OCR engine obtains 89.7% on the held-out subset and 54.7% on the population. For that reason, all comparisons between systems are run on Gold-B.

The third concerns coverage. The format filter required six characters of the national pattern, so anything departing from LNN-DDD fell outside by construction: motorcycle plates, which are common in the region, and also diplomatic, consular and special-series plates. Two consequences follow. The reported accuracy applies to four-wheel vehicles carrying standard plates, and the corpus itself inherits that bias, so a model trained on it would need new data rather than new rules to cover the excluded classes. Widening the filter is not merely a matter of relaxing a regular expression, because that same filter is what prevents spurious detections from entering the silver labels.

The fourth is that the site and the sensor are single. The whole corpus comes from one installation and one camera model, so between-installation variability does not appear in the training data. This is exactly why we measure the cross-optics gap instead of assuming it away, but it also implies that its specific magnitude could differ at another site.

The fifth is the size of the twilight subset. At *n* = 32, those values are indicative and will not support formal tests.

The sixth affects the detection annotation over the corpus at scale, which is silver: the boxes were obtained automatically. The detector comparison in Section 4.6 now uses an entity-grouped split with no plate or burst crossing partitions, so those figures are no longer optimistic in that respect, but the in-domain quality of the 579,292 crops themselves rests on automatic localization and must be read with that caveat.

One last limitation is of a different nature. Recognition ground truth depends on access to the official vehicle registry, which limits how far the verified subset can grow. And because the license plate is personal data, the dataset is distributed under a use agreement rather than in open access, which adds friction for anyone seeking to reproduce these results.

## 7. Conclusions and Future Work

A general-purpose OCR engine, corrected with deterministic rules that encode the national convention, serves as a teacher for distilling compact recognizers that are robust to plate size and to lighting condition and that need no post-processing at inference. Subsequent fine-tuning, on a small verified set chosen by active selection, corrects the teacher’s systematic biases and leaves the recognizer far above it: 91.9% against 54.7% on arbitrary plates, with 0.97 M parameters and a deployable model of 3.7 MB.

The most transferable methodological result is that the composition of the evaluation set determines the conclusion. Over accesses sampled by frequency, dominated by habitual, well-photographed vehicles with labels consolidated by consensus, the teacher looks almost as good as the student: 95.4% against 97.4%. Over arbitrary plates, the difference is 37.2 points. Both figures are correct and answer different questions. Reporting only the first would have understated the contribution of the method by a factor of nineteen, which is why we publish both ground truths while stating which question each one answers.

A second methodological result of the same kind appears along the preprocessing axis. An earlier comparison between architectures, made with each model at its nominal resolution, indicated that the convolutional recurrent network beat the purely convolutional one after fine-tuning. Once the input is equalized and the run repeated across three seeds, that inversion disappears: LPRNet leads under both supervision regimes, with nine-times fewer parameters. Comparing architectures without controlling the input resolution can, therefore, reverse the conclusion, just as doing so without declaring the sampling can.

On the sensing-system side, we document a 465-day continuous deployment whose edge processing keeps images from leaving the institutional network. And we quantify a limitation that is usually assumed rather than measured: a detector trained on a single camera retains 17% of its performance against mobile-device photography, a gap that a merged dataset narrows by raising recall from 0.39 to 0.87 without degrading performance at the original installation.

### Future Work

Several lines remain open. The most immediate concerns the teacher. Replacing the hard deterministic substitution with soft probabilistic filtering, in which per-character confidence from the OCR engine feeds a beam search constrained by the national pattern, would let the pipeline abstain on low-confidence characters instead of committing to a rule-driven guess, and would plausibly reduce the silver-label noise that Section 5.3 identifies as what the larger architecture spends its capacity fitting. Distillation with multiple teachers and format-constrained decoding ought to attack the residual errors of the three-character prefix, where between 80% and 86% of positional failures concentrate. Extension to motorcycle and special-series plates first requires redesigning the capture validation criterion, which currently excludes them. Evaluating attention-based or state-space recognizers under the two supervision regimes described here would establish whether the advantage of the compact architecture holds against other families and how many seeds are needed to tell them apart. Temporal voting also remains to be measured: deciding by majority within a single pass should absorb part of the localization error, although the thin redundancy reported in Section 5.7, a median of one retained crop per pass, limits how much it can contribute. Finally, moving detection onto the camera and measuring end-to-end latency and energy on embedded hardware [9] are the deployment steps this work does not report, because detection and recognition were evaluated separately.

## Figures and Tables

**Table 1:** Specifications of the sensing subsystem and of the capture.

Parameter	Value
Sensor	Hikvision DS-2CD2683G2-LIZS2U, 8 MP, motorized varifocal
Mounting height/angle	2.50 m/<45° to the horizontal
Working resolution/rate	1280 × 720 px/10 fps, constant bit rate
Transport	RTSP (port 554) over a wired local network
Site	Vehicle entrance, Peruvian altiplano (>3800 m a.s.l.)
Operating period	7 March 2025 to 15 June 2026 (465 consecutive days)
Median plate area	5136 px^2^ (q1 4095; q3 6510)
Median crop (width × height)	101 × 51 px

**Table 2:** Corpus partitioning, grouped by plate and stratified by plate type.

Partition	Plates	Frames	% of Frames
Training	23,943	459,598	79.34
Validation	2993	53,266	9.20
Test	2992	66,428	11.47
Total	29,928	579,292	100

**Table 3:** Evaluation sets and their role in the design. The unit of analysis is the crop; in parentheses, the number of distinct plates that compose it.

Set	n (Plates)	Sampling and Role
Gold-A	782 (524)	Sampled in proportion to pass frequency (by-event view), so that a habitual vehicle contributes up to ten crops. Estimates accuracy per logged access. Also used for epoch selection during fine-tuning.
Gold-B	1130 (1074)	One crop per plate except for 53 of them, which contribute between two and four (by-plate view). Reproduces the frequency distribution of the corpus. Drawn from the validation and test partitions. Includes 269 nighttime crops and 32 twilight crops by deliberate oversampling. Main evaluation.
Held-out	689 (667)	Subset of Gold-B containing no plate present in the fine-tuning set, the epoch-selection set or the base training set. Leakage control.

**Table 4:** Systems compared (levels of the “system” factor). n/a: the system has no trainable parameters.

System	Parameters	Description
PaddleOCR raw	n/a	General-purpose OCR engine without domain adaptation [40]. Reference level.
PaddleOCR + corrector	n/a	The above followed by the deterministic position-aware corrector. Acts as the teacher of the distillation.
CRNN	8.73 M	Convolutional recurrent network with CTC loss [27,28], distilled from silver labels.
LPRNet	0.97 M	Purely convolutional network [30,31], distilled from the same labels.
LPRNet fine-tuned	0.97 M	The above, subjected to fine-tuning on actively selected verified ground truth.
CRNN fine-tuned	8.73 M	The distilled CRNN under the same fine-tuning. Allows both architectures to be compared under clean supervision.

**Table 5:** Disjoint roles of the sets during fine-tuning.

Set	Role	Involved in
1850 gold crops	Fitting	Gradient
Gold-A (782)	Epoch selection	Decides which checkpoint is kept; its figure is not reported
Held-out (689)	Leakage control	Measured once, with the epoch already chosen

**Table 6:** Training and inference configuration of the recognizers.

Parameter	Distillation	Fine-Tuning
Data	100,000 silver crops	1850 verified crops
Epoch selection	10,000 validation crops	Gold-A (782)
Optimizer	Adam	Adam
Learning rate	10^−3^, cosine annealing	3 × 10^−4^, constant
Batch size	256	64
Epochs	10	25
Loss	CTC, 37 classes	CTC, 37 classes
Seeds	3	3

**Table 7:** Exact match and CER on the two ground truths, with a unified input of 32 × 100 px. The four trained models are reported as the mean and standard deviation of three seeds; the two PaddleOCR systems are deterministic. Gold-B is the main evaluation.

System	Gold-B (*n* = 1130)		Gold-A (*n* = 782)	
	Exact	CER	Exact	CER
LPRNet + fine-tuning	91.89 ± 0.57	0.0155	97.36 ± 0.07	0.0046
CRNN + fine-tuning	89.73 ± 1.48	0.0202	97.23 ± 0.27	0.0046
LPRNet (silver)	83.54 ± 0.84	0.0310	92.50 ± 0.66	0.0135
CRNN (silver)	79.65 ± 1.51	0.0416	90.49 ± 0.85	0.0170
PaddleOCR + corrector	54.69%	0.0922	95.40%	0.0098
PaddleOCR raw	48.94%	0.1128	65.09%	0.0646

**Table 8:** Post hoc tests on Gold-B, with the reference seed. b10/b01 = discordant plates favoring A/favoring B. n.s. = not significant after Holm.

A vs. B	Δ (pp)	b10/b01	*p* (Holm)
LPRNet-ft vs. PaddleOCR raw	+43.19	519/31	4.0 × 10^−114^
CRNN-ft vs. PaddleOCR raw	+42.39	514/35	4.0 × 10^−109^
LPRNet-ft vs. PaddleOCR + corr	+37.43	460/37	7.4 × 10^−93^
CRNN-ft vs. PaddleOCR + corr	+36.64	455/41	2.3 × 10^−88^
LPRNet vs. PaddleOCR raw	+33.81	427/45	4.1 × 10^−78^
CRNN vs. PaddleOCR raw	+31.15	414/62	6.2 × 10^−64^
LPRNet vs. PaddleOCR + corr	+28.05	369/52	5.2 × 10^−59^
CRNN vs. PaddleOCR + corr	+25.40	356/69	8.9 × 10^−47^
LPRNet-ft vs. CRNN	+12.04	152/16	3.8 × 10^−28^
CRNN-ft vs. CRNN	+11.24	146/19	1.1 × 10^−24^
LPRNet-ft vs. LPRNet	+9.38	118/12	2.4 × 10^−22^
CRNN-ft vs. LPRNet	+8.58	125/28	2.4 × 10^−15^
PaddleOCR + corr vs. raw	+5.75	65/0	2.2 × 10^−19^
LPRNet vs. CRNN	+2.65	99/69	0.050
LPRNet-ft vs. CRNN-ft	+0.80	32/23	0.281 (n.s.)

**Table 9:** Wald tests on the factorial model. Binomial generalized estimating equations (GEE) with an independence structure, equivalent to a GLM with robust errors clustered by plate. 6780 observations from 1130 crops.

Term	χ^2^	df	*p*
System	16.69	5	0.0051
Plate frequency	26.06	3	9.3 × 10^−6^
Lighting class	1.45	2	0.483
System × frequency	49.05	15	1.7 × 10^−5^
System × lighting	34.15	10	1.8 × 10^−4^

**Table 10:** Exact match on Gold-B by number of vehicle passes. Mean of three seeds for the trained models.

System	1–2	3–9	10–49	50+
	(*n* = 539)	(*n* = 290)	(*n* = 196)	(*n* = 105)
LPRNet + fine-tuning	87.8%	94.6%	97.1%	95.9%
CRNN + fine-tuning	85.8%	92.2%	94.0%	94.9%
LPRNet (silver)	79.5%	83.7%	90.6%	90.8%
CRNN (silver)	74.5%	80.2%	88.1%	88.9%
PaddleOCR + corrector	40.4%	57.6%	71.9%	87.6%
PaddleOCR raw	36.4%	50.7%	62.2%	83.8%

**Table 11:** Exact match on Gold-A by plate-area quartile. Mean of three seeds for the trained models.

System	Q1	Q2	Q3	Q4
	≤4324 px^2^ (*n* = 193)	≤4802 (*n* = 194)	≤5616 (*n* = 189)	>5616 (*n* = 206)
LPRNet + fine-tuning	98.1%	99.5%	97.5%	94.5%
CRNN + fine-tuning	97.1%	99.1%	97.2%	95.6%
LPRNet (silver)	92.4%	95.5%	94.7%	87.7%
CRNN (silver)	88.8%	94.3%	92.1%	87.1%
PaddleOCR + corrector	94.3%	97.4%	96.8%	93.2%
PaddleOCR raw	45.1%	73.2%	73.5%	68.4%

**Table 12:** Exact match on Gold-B by lighting class, determined from actual solar elevation. Mean of three seeds for the trained models.

System	Day	Twilight	Night	Δ Day → Night
	(*n* = 829)	(*n* = 32)	(*n* = 269)	
LPRNet + fine-tuning	92.0%	94.8%	91.2%	−0.8
CRNN + fine-tuning	89.7%	92.7%	89.5%	−0.2
LPRNet (silver)	85.9%	79.2%	76.7%	−9.2
CRNN (silver)	80.9%	67.7%	77.1%	−3.8
PaddleOCR + corrector	58.6%	59.4%	42.0%	−16.6
PaddleOCR raw	52.4%	56.2%	37.5%	−14.9

**Table 13:** Effect of fine-tuning on Gold-B and on the held-out subset. Mean and standard deviation of three seeds.

Model	Gold-B (*n* = 1130)		Held-Out (*n* = 689)	
	Exact	CER	Exact	CER
LPRNet, silver	83.54 ± 0.84	0.0310	89.55 ± 0.44	0.0198
LPRNet + fine-tuning	91.89 ± 0.57	0.0155	92.50 ± 0.55	0.0141
Δ	+8.35		+2.95	
CRNN, silver	79.65 ± 1.51	0.0416	85.53 ± 0.69	0.0294
CRNN + fine-tuning	89.73 ± 1.48	0.0202	89.89 ± 1.76	0.0186
Δ	+10.08		+4.36	

**Table 14:** Exact match on Gold-B by architecture and supervision regime, with three seeds and a unified input of 32 × 100 px.

Architecture and Regime	Mean	SD	Parameters	ONNX
LPRNet, silver labels	0.8354	0.0084	0.97 M	3.7 MB
CRNN, silver labels	0.7965	0.0151	8.73 M	35 MB
LPRNet + fine-tuning	0.9189	0.0057	0.97 M	3.7 MB
CRNN + fine-tuning	0.8973	0.0148	8.73 M	35 MB

**Table 15:** Ablation of the ROI resolution, with a single seed and silver labels; hence the figures at 32 × 100 differ from the three-seed mean of Table 7. The median native aspect ratio of the Peruvian plate is 1.98. LPRNet rows marked with † are not interpretable (see text).

Model	Input	Aspect	T	Exact	ms/img
LPRNet	24 × 94	3.92	23	84.42%	0.47
LPRNet	32 × 100	3.12	25	85.22%	0.47
LPRNet †	48 × 96	2.00	24	13.19%	0.48
LPRNet †	64 × 128	2.00	32	5.22%	0.93
LPRNet †	80 × 160	2.00	40	3.81%	0.43
CRNN	24 × 94	3.92	22	80.62%	1.68
CRNN	32 × 100	3.12	24	79.73%	1.88
CRNN	48 × 96	2.00	23	80.35%	1.71
CRNN	64 × 128	2.00	31	80.97%	2.11
CRNN	80 × 160	2.00	39	80.27%	2.41

**Table 16:** Inter-annotator agreement on a subsample of 200 plates from Gold-A.

Comparison	Per Plate	Per Character	Cohen’s κ
Assessor A ↔ Assessor B	99.0%	99.8%	0.998
Assessor A ↔ official registry	92.0%	98.6%	0.985
Assessor B ↔ official registry	93.0%	98.8%	0.987

**Table 17:** Detection comparison over the ten architectures trained under identical conditions, on the entity-grouped split with no leakage. The last two columns give the figure obtained on the original file-level random split and the difference, so that the effect of leakage on each architecture is visible. Rows are ordered by mAP@50–95 on the clean split.

Model	mAP@50–95	mAP@50	Recall	ms/img	mAP@50–95 (Leaky Split)	Δ (pp)
YOLOv8s	0.9331	0.9950	0.9993	2.18	0.9451	−1.20
YOLOv11m	0.9327	0.9950	1.0000	4.98	0.9397	−0.70
YOLOv11s	0.9326	0.9950	1.0000	2.16	0.9373	−0.47
YOLOv12n	0.9258	0.9950	1.0000	1.30	0.9320	−0.62
YOLOv8m	0.9207	0.9950	1.0000	4.99	0.9428	−2.21
YOLOv12s	0.9206	0.9950	1.0000	2.56	0.9325	−1.19
YOLOv8n	0.9201	0.9950	1.0000	1.18	0.9303	−1.02
YOLOv11n	0.9192	0.9950	1.0000	1.27	0.9260	−0.68
YOLOv12m	0.9053	0.9950	1.0000	5.86	0.9331	−2.78
RT-DETR-l	0.8805	0.9947	0.9974	13.63	0.9110	−3.05

**Table 18:** Performance of the single-camera detector inside and outside its optical domain. Both columns come from a single paired evaluation with its own test partitions, distinct from the 260 images of Table 17, hence the small difference in the in-domain figure.

Metric	In-Domain	Cross-Domain	Retention
mAP@50–95	0.908	0.157	17.3%
mAP@50	0.988	0.386	39.0%
Precision	0.978	0.610	62.3%
Recall	0.997	0.388	39.0%

## Data Availability

The data supporting the results of this study are deposited on Zenodo under restricted access, DOI 10.5281/zenodo.22021714 (https://doi.org/10.5281/zenodo.22021714). The deposit contains the 579,292 plate crops with their labels, the three plate-grouped partitions, the Gold-A and Gold-B evaluation sets, and the 2600 images with manually annotated localization. The restriction reflects the fact that a license plate constitutes personal data linkable to its holder through the official vehicle registry, and its processing is governed by Peruvian personal data protection law (Ley 29733). Access is granted upon a reasoned request through the repository itself, for academic or non-commercial research use, and subject to acceptance of a use agreement that prohibits re-identification of holders and redistribution of the data. The deposit is limited to crops of the license plate region, with a single exception: the 2600 images of the detection ground truth are complete frames, since training a detector requires the context of the scene. They are deposited after the automatic face sweep and blurring described in Section 3.3.4. No other complete frame is published. The code for dataset generation, training, evaluation, statistical testing and figures is openly available at https://github.com/AngelDX/peruvian-alpr (accessed on 14 October 2026) under the MIT license.

## References

[1] Quraishi A.A., Feyzi F., Shahbahrami A. (2024). Detection and recognition of vehicle licence plates using deep learning in challenging conditions: A systematic review. Int. J. Intell. Syst. Technol. Appl..

[2] Jagtap V.H., Dhotre R.V., Khandare U.R., Khuspe H.N., Kokare R.B. (2024). Automatic License Plate Recognition System: A Systematic Survey. J. Eng. Technol. Ind. Appl..

[3] Ning D., Han D.S. (2026). Deep learning for license plate recognition: A comprehensive survey of datasets, methods, and future directions. ICT Express.

[4] Tippannavar S.S., Yashwanth S. (2023). Real-Time Vehicle Identification for Improving the Traffic Management system-A Review. J. Trends Comput. Sci. Smart Technol..

[5] Kamarozaman M.H.B., Syafeeza A., Wong Y., Hamid N.A., Saad W.H.M., Samad A.S.A. (2024). Enhancing Campus Security and Vehicle Management with Real-Time Mobile License Plate Reader App Utilizing. J. Eng. Sci. Technol..

[6] Yi S.T.J., Saruchi S.A., Helta F., Izni N.A. (2025). Automatic number plate recognition via convolutional neural network for residential gate access control. Int. J. Veh. Inf. Commun. Syst..

[7] Upadhyay A.K., Verma A., Varshney A., Rai A., Verma A., Rajput K., Singh S. (2026). Automatic Vehicle Gate Access Control System Using YOLOv8 and Tesseract Optical Character Recognition. J. Inst. Eng. Ser. B.

[8] Chaturvedi P., Lavingia K., Raval G. (2023). Detection of traffic rule violation in University campus using deep learning model. Int. J. Syst. Assur. Eng. Manag..

[9] Nardes G.B., Rausch T.H., Pereira B.H., Melo D.R., Zeferino C.A. (2026). A resource-constrained CNN accelerator for real-time license plate character recognition on FPGA platforms. Integration.

[10] Satya B., Manongga D., Hendry, Aminuddin A. (2025). Optimized YOLOv8 for Automatic License Plate Recognition on Resource Constrained Devices. Eng. Technol. Appl. Sci. Res..

[11] Ma Z., Wu Z., Cao Y. (2023). End-to-End Light License Plate Detection and Recognition Method Based on Deep Learning. Electronics.

[12] Cho H., Kim J., Jun K. (2024). Lightweight Adversarial Domain Adaptive Model for License Plate Detection. J. Korean Inst. Commun. Inf. Sci..

[13] Mulia D.A., Safitri S., Negara I.G.P.K. (2024). YOLOv8 and Faster R-CNN Performance Evaluation with Super-resolution in License Plate Recognition. Int. J. Comput. Digit. Syst..

[14] Kabiraj A., Pal D., Ganguly D., Chatterjee K., Roy S. (2023). Number plate recognition from enhanced super-resolution using generative adversarial network. Multimed. Tools Appl..

[15] Ding H., Gao J., Yuan Y., Wang Q. (2024). FF-LPD: A Real-Time Frame-by-Frame License Plate Detector with Knowledge Distillation and Feature Propagation. IEEE Trans. Image Process..

[16] Tian C., Zhang X., Liang X., Li B., Sun Y., Zhang S. (2023). Knowledge Distillation with Fast CNN for License Plate Detection. IEEE Trans. Intell. Veh..

[17] Silva S.M., Jung C.R. (2022). A Flexible Approach for Automatic License Plate Recognition in Unconstrained Scenarios. IEEE Trans. Intell. Transp. Syst..

[18] Lee S., Park G. (2026). A Comparative Study of OCR Architectures for Korean License Plate Recognition: CNN–RNN-Based Models and MobileNetV3–Transformer-Based Models. Sensors.

[19] Ismail A., Mehri M., Sahbani A., Essoukri Ben Amara N. (2025). ELP 1.0: A Comprehensive and Geographically Diverse Dataset of European License Plates. IEEE Trans. Intell. Transp. Syst..

[20] Zhang Y., Chen J., Song R., Liu Y., Behera A., Zhang M., Zhang W. (2025). SLPDR: A Benchmark for Ship License Plate Detection and Recognition. IEEE Trans. Intell. Transp. Syst..

[21] Ruseno N., Ernawan F., Salamat M.A., Arshad M.S., Amnur H. (2026). An Enhanced YOLOv8 Model Integrated with CNN for Real-Time Vehicle License Plate Recognition. Int. J. Inform. Vis..

[22] Zhang X., Yu S. (2025). A Lightweight License Plate Recognition Method Based on YOLOv8. Electronics.

[23] Al-batat R., Angelopoulou A., Premkumar S., Hemanth J., Kapetanios E. (2022). An End-to-End Automated License Plate Recognition System Using YOLO Based Vehicle and License Plate Detection with Vehicle Classification. Sensors.

[24] Zhu S., Wang Y., Wang Z. (2024). A lightweight license plate detection algorithm based on deep learning. IET Image Process..

[25] Lei J., Zhang Z., Qian X., Wang W., Yang S. (2025). Improved Lightweight Multi-Directional License Plate Detection Algorithm of YOLOX. Comput. Eng. Appl..

[26] Ding H., Gao J., Yuan Y., Wang Q. (2024). An End-to-End Contrastive License Plate Detector. IEEE Trans. Intell. Transp. Syst..

[27] Dang L.T.A., Ngoc V.D., Thien Vu P.C.L., Truong N.N., Bao P.T., Trinh T.D. (2024). Vietnam Vehicle Number Recognition Based on an Improved CRNN with Attention Mechanism. Int. J. Intell. Transp. Syst. Res..

[28] Hatami S., Jamali F.S., Sadedel M. (2024). Iranian license plate recognition using a reliable deep learning approach. Sci. Iran..

[29] Deng J., Wei H., Lai Z., Gu G., Chen Z., Chen L., Ding L. (2023). Spatial Transform Depthwise Over-Parameterized Convolution Recurrent Neural Network for License Plate Recognition in Complex Environment. J. Comput. Inf. Sci. Eng..

[30] Luo S., Liu J. (2022). Research on Car License Plate Recognition Based on Improved YOLOv5m and LPRNet. IEEE Access.

[31] Zou Y., Zhang Y., Yan J., Jiang X., Huang T., Fan H., Cui Z. (2022). License plate detection and recognition based on YOLOv3 and ILPRNET. Signal Image Video Process..

[32] Seo T.M., Kang D.J. (2022). A Robust Layout-Independent License Plate Detection and Recognition Model Based on Attention Method. IEEE Access.

[33] Dittakan K., Thaenthong J., Prasomkit T. (2025). A Comparative Study on Thai License Plate Recognition: Object Detection and Transformer Learning Approaches. IEEE Access.

[34] Issaoui S., Alzakari S.A., Saidi I., Ejbali R., Serir A. (2026). Hierarchical Vision–Language Fusion with Structural Constraint Reasoning for Robust Multi-Jurisdiction License Plate Recognition. Appl. Sci..

[35] Pan S., Chen S.B., Luo B. (2023). A super-resolution-based license plate recognition method for remote surveillance. J. Vis. Commun. Image Represent..

[36] Huang D.Y., Chen C.H., Chen T.Y., Hung K.L., Wen C.K. (2026). Cost-Effective Restoration of Motion-Blurred Vehicle License Plates in Daytime and Nighttime Scenarios. IEEE Trans. Consum. Electron..

[37] Mohammed M.J., Kadhm M.S., Al-Hamadani A.A., Zaben S.O. (2026). Proposed Iraqi License Plates Detection System Using LAB Color Space, CNN and YOLOv8-Seg Models. Ing. Syst. Inf..

[38] Zhao K., Chang Y., Wu W., Luo H., Li Z., He S., Guo D. (2023). Efficient memory reuse methodology for CNN-based real-time image processing in mobile-embedded systems. J. Real-Time Image Process..

[39] Swati, Kawa S.D., Kamble S., Desai D., Karelia P.H., Engineer P. (2024). An Efficient Deep Learning based License Plate Recognition for Smart Cities. Electron. Lett. Comput. Vis. Image Anal..

[40] Buleu B., Robu R., Filip I. (2025). A Deep Learning-Based System for Automatic License Plate Recognition Using YOLOv12 and PaddleOCR. Appl. Sci..

